# The EV-mitochondrial outsourcing network as a therapeutic target for age-related testosterone deficiency: from network collapse to clinical intervention

**DOI:** 10.3389/fendo.2026.1866059

**Published:** 2026-06-22

**Authors:** Lu Zhou, Xingzhao Tian, Xiteng Wang, Xiucheng Lan, Meijing Wang, Junjun Li, Liang Dong, Xujun Yu, Fang Yang

**Affiliations:** 1 Chengdu University of Traditional Chinese Medicine, Chengdu, Sichuan, China; 2 Chengdu Fifth People’s Hospital, Chengdu, Sichuan, China; 3 The Second Affiliated Hospital of Chengdu University of Traditional Chinese Medicine, Chengdu, Sichuan, China

**Keywords:** extracellular vesicles, late-onset hypogonadism, leydig cells, mitochondrial transfer, reproductive aging, testicular macrophages

## Abstract

The longevity and functional maintenance of Leydig cells (LCs) depend on an extracellular vesicle (EV)–mediated mitochondrial outsourcing system. In contrast to the conventional focus on intracellular quality control, LCs orchestrate a bidirectional EV transfer network with testicular macrophages: they export EVs containing defective mitochondria to CD206^+^ macrophages for clearance (the scavenger pathway), while importing EVs with healthy mitochondria from MHCII^+^ macrophages (the donor pathway). During aging, this network undergoes three-dimensional disruption—donor-side collapse characterized by diminished PGC-1α, a shift toward Drp1 predominance over Mfn2, and NAD^+^ depletion; scavenger-side obstruction reflected in reduced TREM2 and impaired phagosome-lysosome fusion; and communication uncoupling driven by decay of the VCAM1/ITGβ1 axis. These coordinated failures precipitate a mitochondrial quality crisis, steroidogenic enzyme dysfunction, and progressive loss of LCs, thereby initiating and driving late-onset hypogonadism (LOH). Individual variability in EV network efficiency dictates LOH susceptibility, and the point of network decompensation marks the transition from a compensated state to clinical disease. In-depth dissection of this network and its dysregulation may yield novel strategies for early diagnosis (INSL3, EV-miRNA signatures, mtDNA mutation burden) and targeted therapy (MSC-EVs, NAD^+^ precursors, TREM2 activation).

## 1 Introduction: a new paradigm for mitochondrial homeostasis maintenance in leydig cells

Leydig cells (LCs) are the central effector cells of the male reproductive endocrine system, responsible for the synthesis and secretion of testosterone (1). Testosterone not only maintains male secondary sexual characteristics and spermatogenesis but also exerts widespread regulatory effects on systemic metabolic homeostasis, bone mineral density, muscle mass, and cognitive function (2, 3). The steroidogenic process in LCs is a highly energy-dependent biochemical cascade: cholesterol is transported to the inner mitochondrial membrane by the steroidogenic acute regulatory protein (StAR), subsequently converted to pregnenolone by cytochrome P450 cholesterol side-chain cleavage enzyme (CYP11A1), and thereafter undergoes multiple enzymatic reactions within the endoplasmic reticulum to yield testosterone (4, 5). Each of these steps requires an adequate supply of ATP and precise redox regulation, and mitochondria provide the vast majority of ATP demand through oxidative phosphorylation—studies have demonstrated that primary LCs rely almost exclusively on mitochondrial respiration for their energy requirements (6).Unlike most somatic cells, LCs are considered long-lived, terminally differentiated cells with limited proliferative and renewal capacity in adults; their functional maintenance is therefore intimately linked to the lifespan of the individual (7). This biological characteristic imposes a unique metabolic challenge upon LCs: during decades of sustained testosterone synthesis, mitochondria inevitably accumulate oxidative damage. Reactive oxygen species (ROS), by-products of the electron transport chain, are generated in abundance under prolonged high-load conditions, leading to mitochondrial DNA (mtDNA) mutations, protein oxidation, membrane lipid peroxidation, and disruption of mitochondrial dynamics (8). The terminally differentiated state of LCs restricts their ability to dilute damage through cell division, rendering mitochondrial quality control a critical determinant of the functional lifespan of LCs.

Traditional intracellular quality control mechanisms—particularly mitophagy—are responsible for clearing a portion of damaged mitochondria, yet their capacity exhibits inherent limitations. On the one hand, the degradative capacity of mitophagy is finite; when the rate of damage exceeds the clearance threshold, clearance can no longer keep pace with production (9). On the other hand, mitophagy itself consumes energy and biosynthetic resources, and its efficiency declines further under conditions of energy stress or aging (9, 10). These limitations have prompted investigators to re-evaluate the homeostatic maintenance strategies of LCs and to explore the existence of complementary pathways that extend beyond cell-autonomous mechanisms. Notably, LCs are able to maintain testosterone synthesis even in the face of severe mitochondrial dysfunction—in mtDNA mutator mice, the respiratory chain complex activity of LCs is reduced to merely 20% of normal levels, yet circulating testosterone remains completely unaffected, strongly suggesting the presence of a unique compensatory mechanism (11). This observation underscores the necessity of exploring non-cell-autonomous mechanisms of homeostatic maintenance in LCs. Subsequent studies have revealed that LCs can maintain steroidogenesis through several mechanisms (1): Cytochrome b5 bypass—in mtDNA mutator mice, Shabalina et al. demonstrated that LCs upregulate cytochrome b5 three-fold, transferring electrons derived from the pentose phosphate pathway directly to cytochrome c and bypassing impaired respiratory chain Complex III, thereby preserving mitochondrial membrane potential and testosterone synthesis (11); biophysical studies further confirm that direct electron transfer from cytochrome b5 to cytochrome c is thermodynamically feasible (12) (2). Pentose phosphate pathway enhancement—NR5A1 directly regulates PPP-related genes to supply the NADPH reducing equivalents required for CYP450-mediated steroidogenic reactions (13) (3). Lactate shuttle—within the testicular interstitium, Sertoli cells produce abundant lactate, which LCs can import via monocarboxylate transporters as an alternative energy substrate; exogenous lactate directly stimulates testosterone secretion by LCs (14). Although these compensatory mechanisms can transiently sustain testosterone synthesis, they possess inherent capacity limits; chronic overload ultimately leads to metabolic exhaustion and may accelerate the senescence-associated secretory phenotype (SASP) of LCs.

Notably, Leydig cell endocrine function exhibits marked age-distribution characteristics. Epidemiological and longitudinal data indicate that serum total testosterone declines progressively with aging, while free testosterone falls more rapidly, largely reflecting age-associated increases in SHBG (15, 16). The prevalence of LOH rises significantly after age 40; the European Male Aging Study (EMAS) reported rates increasing from 0.1% in men aged 40–49 years to 5.1% in those aged 70–79 years (15), and a national survey in China demonstrated a comparable progressive increase in LOH prevalence across age groups (17). The Baltimore Longitudinal Study of Aging further showed that approximately 50% of men in their 80s fall into the hypogonadal range (16).A cross-sectional study revealed that serum testosterone and LH display bimodal peaks at 13–19 and 30–45 years, whereas INSL3 peaks only during puberty (13–19 years) (18). This age-dependent fluctuation implies that LCs enter a prolonged high-load synthetic phase after puberty, rendering the network more vulnerable to crossing the critical decompensation threshold in middle-to-old age.” (19). Histologically, stereological studies demonstrate that the total number of LCs decreases significantly in older men (20). Concurrently, the intrinsic steroidogenic capacity of individual LCs declines, as evidenced by temporal reductions in testosterone production, StAR expression, and P450 side-chain cleavage enzyme (P450scc) expression during aging (21).

**Figure 1:**
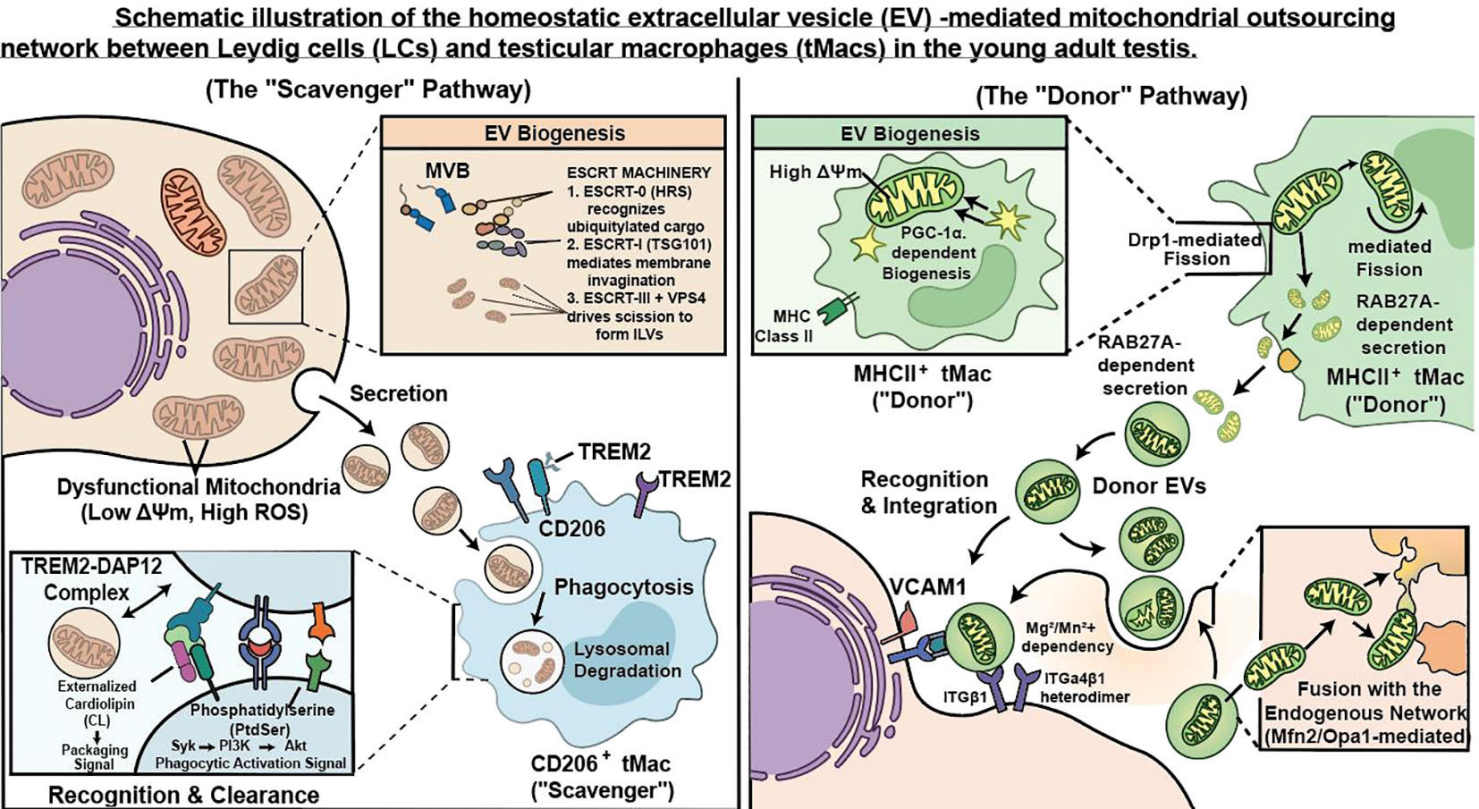
Schematic of the bidirectional EV mitochondrial transfer network in the young state.

The seminal work by Xia et al. provided the first systematic elucidation of the molecular mechanisms underlying bidirectional mitochondrial transfer between LCs and testicular macrophages (tMacs) via extracellular vesicles (EVs). This study revealed the existence of two functionally distinct macrophage subpopulations within the testicular interstitium: macrophages with high CD206 expression (CD206^+^) primarily execute a “scavenger” function, whereas macrophages with high MHC class II molecule expression (MHCII^+^) assume a “donor” role. LCs directionally export EVs containing defective mitochondrial “cargo” to CD206^+^ macrophages for clearance, whilst simultaneously acquiring EVs laden with healthy mitochondria from MHCII^+^ macrophages, thereby achieving highly efficient mitochondrial quality control and functional renewal (22). A schematic overview of this bidirectional EV-mediated mitochondrial transfer network is presented in **Figure 1**. The figure illustrates the two complementary arms of the network—the scavenger pathway, wherein CD206^+^ tMacs recognize and engulf LC-derived EVs containing defective mitochondria via TREM2-DAP12-Syk signaling, and the donor pathway, wherein MHCII^+^ tMacs supply functional mitochondria to LCs through VCAM1-ITGβ1-mediated EV uptake. This discovery has considerably broadened our understanding of the homeostatic maintenance mechanisms of LCs, extending the investigative perspective from isolated cell-autonomous mechanisms to a dynamic intercellular collaborative network.

It must be emphasized that the EV network is not the sole mechanism maintaining LC homeostasis. The hypothalamic-pituitary-gonadal (HPG) axis, as the classical endocrine regulatory pathway, also plays a critical role in LOH pathogenesis. Aging is accompanied by slowed GnRH neuronal pulsatility, reduced LH pulse amplitude, and altered pulse frequency (23). Concurrently, pituitary integration of GnRH drive is impaired at the ensemble level, although individual gonadotropes retain responsiveness to exogenous GnRH (23). At the testicular level, LC exhibit markedly diminished secretory responsiveness to LH (24), attributable to receptor downregulation and cAMP signaling defects, such that prolonged LH exposure cannot restore steroidogenic capacity (25). Furthermore, aging erodes endogenous testosterone-driven negative feedback on pulsatile LH secretion by 40–50% (26), and jointly alters the dose-dependent actions of GnRH through age–testosterone feedback interactions (27). By focusing on the EV-mediated mitochondrial transfer network, this review does not deny the substantial contribution of HPG-axis aging mechanisms; rather, it aims to illuminate a previously underappreciated core pathway that integrates cell-autonomous damage with immune microenvironment dysregulation.

## 2 The EV-mediated bidirectional mitochondrial transfer network in the young state

### 2.1 The scavenger pathway: quality control function of CD206^+^/TREM2

The heterogeneity of testicular macrophages provides the structural foundation for the efficient operation of the EV network. Among them, CD206^+^ macrophages act as the “scavenger” subpopulation, specifically recognizing LCs-derived EVs containing defective mitochondria via high surface expression of triggering receptor expressed on myeloid cells 2 (TREM2) (22, 28). TREM2, a lipid-sensing receptor, forms a signaling complex with the adaptor protein DAP12. Through its intracellular immunoreceptor tyrosine-based activation motif (ITAM), this complex recruits spleen tyrosine kinase (Syk), thereby activating the PI3K-Akt and MAPK signaling pathways and triggering efficient phagocytosis in response to “eat-me” signals, such as phosphatidylserine and cardiolipin, exposed on the EV membrane (29). Concurrently, TREM2 signaling recruits the tyrosine phosphatase SHP-1 to restrain excessive NF-κB activation, thereby maintaining an anti-inflammatory microenvironment whilst facilitating phagocytic clearance (30). Notably, the recognition and phagocytic mechanism mediated by TREM2 is evolutionarily highly conserved, sharing the same molecular logic as macrophage clearance of apoptotic cells (efferocytosis), both relying on the precise sensing of phosphatidylserine on the membrane surface (28). Moreover, TREM2 signal intensity is tightly negatively regulated by the protein tyrosine phosphatases SHP-1 and SHIP-1 to prevent tissue damage caused by excessive phagocytosis (29).

The selective packaging of damaged mitochondria into EVs is a prerequisite for the efficient operation of the scavenger pathway and involves multiple tiers of damage recognition. The dissipation of mitochondrial membrane potential induces the redistribution of cardiolipin from the inner to the outer mitochondrial membrane, serving as a key packaging signal (29, 30); simultaneously, ubiquitylation of outer mitochondrial membrane proteins provides a molecular tag for cargo sorting (31). The dynamic allocation of these damaged mitochondria between autophagic degradation and EV export depends upon the severity of damage and is ultimately executed through the multivesicular body (MVB) pathway. In this process, the ESCRT-0 complex recognizes ubiquitylated substrates, ESCRT-I/II mediate membrane invagination, and ESCRT-III together with VPS4 drive membrane scission to generate intraluminal vesicles (ILVs); among these components, HRS and TSG101 play essential roles in vesicle formation, and human chorionic gonadotrophin (hCG) stimulation enhances MVB biogenesis (32, 33).

Genetic evidence demonstrates that conditional knockout of Trem2 results in markedly reduced clearance efficiency of LCs-derived defective mitochondria, leading to their intracellular accumulation, elevated oxidative stress, increased mtDNA damage, and ultimately a decline in serum testosterone levels of approximately 35% (22). These findings firmly establish the TREM2-mediated scavenger pathway as a central mechanism in maintaining testicular mitochondrial quality and endocrine function.

### 2.2 The donor pathway: energy support via MHCII^+^/VCAM1-ITGβ1

MHCII^+^ macrophages, as the “donor” subpopulation, constitute an energy-support pathway by delivering EVs containing functional mitochondria to LCs. Although this subpopulation possesses a relatively lower absolute mitochondrial mass, it exhibits a distinct “quality-preferred” phenotype: high-resolution imaging reveals that its mitochondria display higher membrane potential, lower ROS levels, and greater ATP synthesis capacity. This functional advantage is attributed to PGC-1α-driven high rates of mitochondrial biogenesis and a fusion-dominated network architecture, which favors the assembly of respiratory chain supercomplexes (22, 34). Indeed, PGC-1α is recognized as a master regulator of mitochondrial biogenesis and also participates in the precise regulation of mitochondrial dynamics (fusion/fission) and mitophagy (35). Notably, the function of PGC-1α extends beyond biogenesis; it also coordinates mitochondrial metabolism with anti-inflammatory gene expression through the NAD^+^-SIRT1/SIRT3 axis, constituting a regulatory hub that links metabolic status to immune function (35).

As the initiating point of the donor pathway, this subpopulation responds to demand signals from LCs by generating small mitochondrial fragments suitable for packaging through Drp1-mediated mitochondrial fission and promotes EV secretion via the RAB27A/RAB35-dependent MVB trafficking pathway (36). The uptake of donor EVs by LCs depends upon the specific recognition between integrin β1 (ITGβ1) and vascular cell adhesion molecule 1 (VCAM1), an interaction that requires the participation of divalent cations such as Mg²^+^ or Mn²^+^ to form stable adhesive contacts (37). Genetic evidence from knockout models further substantiates the indispensability of this pathway: LC-specific Vcam1 knockout leads to a reduction in EV uptake efficiency exceeding 70%, severely impaired integration of exogenous mitochondria, a decrease in ATP production of approximately 40%, and a reduction in testosterone secretion of 45–50% (22). Single-cell transcriptomic analysis in mice further reveals that the interaction strength between testicular macrophages and LCs via the Vcam1–Itga4/Itgb1 axis markedly declines with aging (notably reduced by 24 months) (38). Although the single-cell dynamics of this pathway in aging human testes remain to be directly validated, the functional necessity of VCAM1–α4β1 interactions in testicular interstitial tissue reconstruction has been established in mammalian models (39). These data robustly demonstrate that functional mitochondrial transfer mediated by the VCAM1–ITGβ1 axis—with ITGα4 serving as an obligate heterodimeric partner—within the MHCII^+^ subpopulation constitutes a critical mechanism for maintaining LC energy metabolism and endocrine function, but remains to be directly validated in human testes.

Altogether, the EV-mediated bidirectional mitochondrial transfer network achieves efficient renewal of the LC mitochondrial pool through a collaborative “in-and-out” mechanism. In normal adult mice, a substantial proportion of mitochondria undergo daily turnover via this pathway, at a rate significantly exceeding that observed in other terminally differentiated cells. This network continuously clears damaged mitochondria whilst importing exogenous healthy ones, thereby alleviating the biosynthetic burden on LCs and maintaining steroidogenesis in an optimal state. Single knockout of Trem2 or Vcam1 results in a 30–40% reduction in testosterone synthesis, whereas double knockout produces a decline exceeding 70%, demonstrating a pronounced dose effect. It is worth emphasizing that this functional redundancy may be regarded as an evolutionarily adaptive strategy underpinning LC longevity: when one pathway is compromised, the other can still provide a degree of compensation, affording a valuable therapeutic window for potential clinical intervention.

## 3 Aging-induced three-dimensional collapse of the EV network: from compensation to decompensation

**Figure 2:**
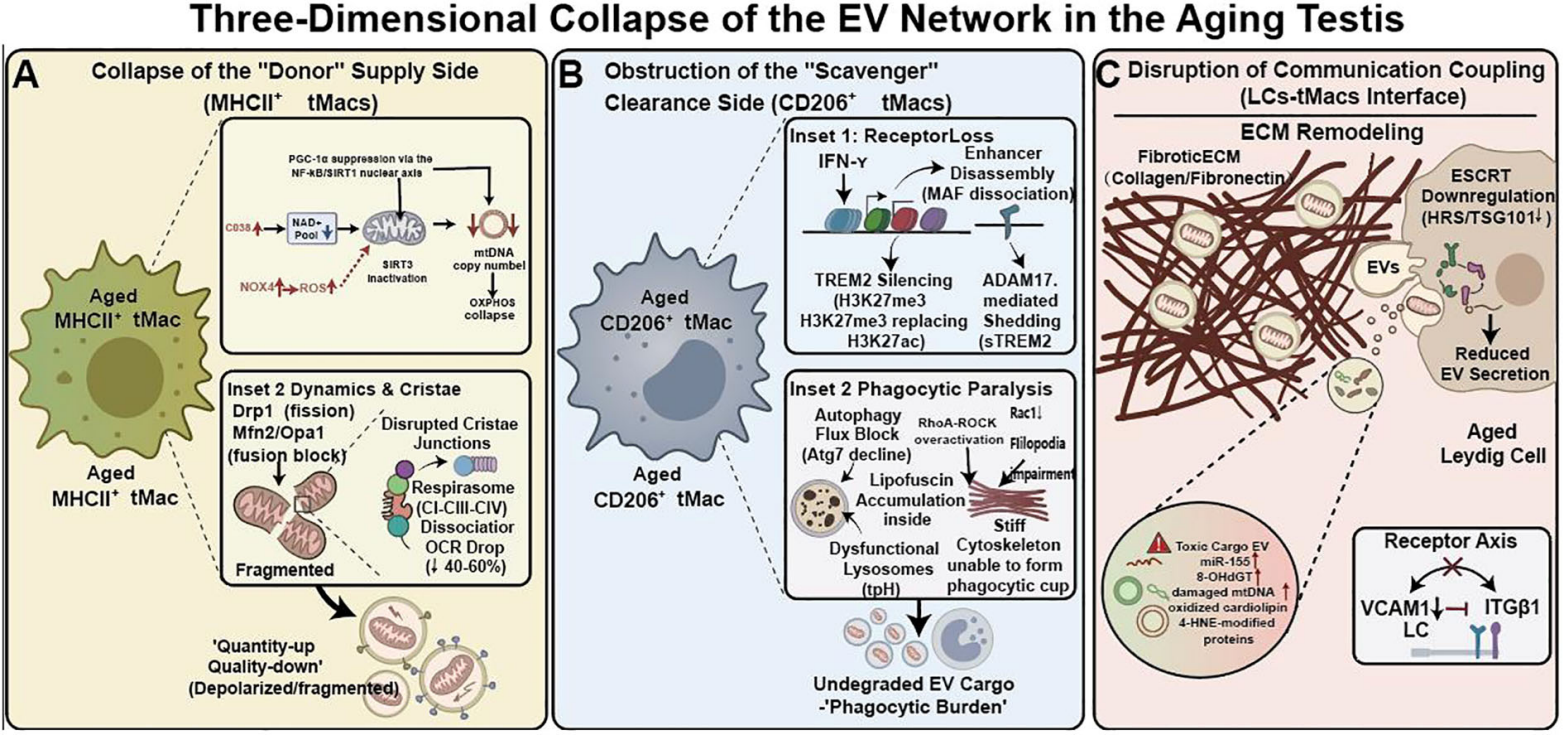
Three-dimensional network collapse in the aging testis. **(A)** Supply-side collapse in MHCII+ donor macrophages: PGC-1α suppression, Drp1-mediated mitochondrial fission, cristae disruption, and OXPHOS collapse. **(B)** Clearance-side obstruction in CD206+ scavenger macrophages: TREM2 epigenetic silencing and shedding, autophagic-lysosomal dysfunction, and RhoA-ROCK-mediated cytoskeletal rigidity. **(C)** Communication decoupling: impaired EV biogenesis and toxic cargo transformation, VCAM1–ITGβ1/α4 axis decline, and ECM remodeling.

aging is the predominant risk factor for late-onset hypogonadism (LOH), yet not all aging men progress to clinical LOH. The possibility of this individual variation lies in the differential decline of EV network efficiency. Specifically, aging systematically impairs the EV network through three dimensions—”supply-side collapse”, “clearance-side obstruction”, and “communication decoupling”—and the tipping point of network decompensation marks the critical phase transition of the disease from the “compensated stage” to the “clinical stage”. **Figure 2** provides a mechanistic overview of the three-dimensional network collapse in the aging testis. The figure depicts how aging systematically impairs the EV outsourcing network at three levels.

### 3.1 Supply-side collapse: functional failure of the donor subpopulation

Within the EV-mediated bidirectional mitochondrial transfer network, MHCII^+^ macrophages, as the “donor” subpopulation, bear the critical responsibility of supplying functional mitochondria to LCs. In the young state, this subpopulation continuously produces “high-quality” mitochondria for EV packaging and transfer through active PGC-1α-driven mitochondrial biogenesis, a finely tuned fusion/fission dynamic balance, and an efficient OXPHOS system (22, 34). However, the aging process systematically erodes the material basis and functional efficiency of this supply pathway at multiple levels. It is important to emphasize that this “supply-side collapse” is not the consequence of a single molecular event but rather a multi-tiered metabolic restructuring driven by the aging microenvironment—extending from blocked mitochondrial biogenesis governed by nuclear genome regulation, through imbalanced dynamics at the organelle level, ultimately culminating in the comprehensive failure of the OXPHOS system.

#### 3.1.1 Transcriptional inactivation of mitochondrial biogenesis

The ability of MHCII^+^ macrophages to maintain a high-quality mitochondrial pool depends primarily on their active mitochondrial biogenesis, the central regulatory hub of which is the transcriptional coactivator PGC-1α. By activating downstream transcription factors such as NRF-1/2 and ERRα, PGC-1α coordinately drives the expression of nuclear-encoded mitochondrial proteins and the replication of mtDNA, functioning as the “master switch” of mitochondrial biogenesis (11, 21). However, male reproductive aging is accompanied by systemic alterations in the testicular microenvironment, among which chronic low-grade inflammation (inflammaging) and the progressive accumulation of oxidative stress are hallmark features. Basal levels of pro-inflammatory cytokines (IL-1β, IL-6, TNF-α) and chemokines (CCL2, CXCL10) are elevated in the aged testis, creating a low-grade but persistent inflammatory state. The sources of this inflammatory milieu are multifaceted: the senescence-associated secretory phenotype (SASP) of aged LCs and Sertoli cells, infiltration of systemic inflammatory factors due to increased permeability of the blood-testis barrier, and altered activation states of testis-resident immune cells (40, 41).

Against this backdrop, aging-related chronic low-grade inflammation directly suppresses the PGC-1α pathway through pro-inflammatory cytokines. As a core node linking oxidative stress, mitochondrial metabolism, and inflammatory responses, PGC-1α not only drives mitochondrial biogenesis via NRF-1/2 and ERRα but also coordinately upregulates the expression of antioxidant genes such as MnSOD, catalase, and Trx2. Under inflammatory conditions, diminished PGC-1α levels compromise antioxidant defenses and precipitate oxidative stress, which in turn promotes NF-κB activation and establishes a self-amplifying vicious cycle wherein low PGC-1α begets elevated reactive oxygen species, which further drives NF-κB activation. Within the testicular microenvironment, aging-related chronic low-grade inflammation transcriptionally represses Ppargc1a through the NF-κB pathway, thereby entrapping MHCII^+^ donor macrophages in a state of sustained PGC-1α functional suppression. defense The consequences include not only impaired mitochondrial biogenesis but also a comprehensive weakening of the antioxidant defense system—a double blow that collectively constitutes the molecular basis of donor-side collapse (42).

Kang et al. utilized a telomere dysfunction mouse model to demonstrate that PGC-1α and the ubiquitin-editing enzyme TNFAIP3 coordinately regulate macrophage mitochondrial metabolism and inflammatory gene expression. It should be noted that this model did not directly validate the corresponding mechanisms in testicular macrophages, and the following inferences rely on cross-tissue analogy. Telomere damage can lead to mitochondrial abnormalities, oxidative stress, and excessive activation of the NLRP3 inflammasome through the p53–PGC-1α pathway (43). Specifically, telomere shortening activates p53, which can directly bind to the promoter region of the Ppargc1a gene and inhibit its transcriptional initiation, thereby directly translating genomic instability signals into mitochondrial metabolic impairment. Notably, systematic modeling studies have further revealed that the responsiveness of the AMPK–NAD^+^–PGC-1α–SIRT1 signaling axis to stimuli is significantly diminished during aging (44). The age-related decline in NAD^+^ levels leads to insufficient deacetylase activity of SIRT1, which can no longer effectively antagonize the inhibitory acetylation of PGC-1α mediated by GCN5, resulting in an “acetylation lock” state. Under this condition, even in the presence of AMPK activation signals, PGC-1α cannot escape transcriptional repression, causing mitochondrial biogenesis to completely lose its adaptive upregulation capacity in response to external demand signals, such as the energy requirements of LCs (45).

The functional inhibition of PGC-1α signaling in aged macrophages has profound metabolic consequences. Studies have shown that the mRNA and protein levels of various mitochondrial respiratory chain complex subunits are generally decreased in aged macrophages, and mtDNA copy number is reduced by 30–50%; these changes can all be attributed to the attenuation of PGC-1α activity (43). The suppression of PGC-1α in aged MHCII^+^ donor macrophages may produce a “dual transcriptional–translational checkpoint” effect: on the one hand, it reduces the transcription of both nuclear- and mtDNA-encoded mitochondrial genes; on the other, it impairs the translational efficiency of already transcribed mRNAs, thereby hindering the assembly of respiratory chain complexes due to insufficient subunit supply. This translational-level impairment acts synergistically with the hyperacetylation of respiratory chain complexes resulting from SIRT3 inactivation, together constituting the molecular basis of donor-side OXPHOS collapse (46).

#### 3.1.2 Mitochondrial dynamics imbalance and network fragmentation

Healthy functional mitochondrial transfer requires not only a sufficient quantity of mitochondria but also appropriate mitochondrial morphology for packaging. Mitochondria maintain network integrity through a dynamic balance of fusion and fission—fusion (mediated by Mfn1/2 and OPA1) enables mitochondria to form interconnected networks for sharing metabolites and mtDNA, whereas fission (mediated by Drp1) generates small mitochondrial fragments suitable for EV encapsulation (34, 40). In young MHCII^+^ macrophages, this dynamic balance is precisely regulated, ensuring both the functional integrity of the mitochondrial network and the ability to generate transferable mitochondrial fragments through moderate fission in response to LCs’ demands.

However, aging tilts this balance toward pathological fragmentation. In aged macrophages, the expression of the pro-fission protein Drp1 is upregulated, whereas the expression of the pro-fusion proteins Mfn2 and OPA1 is significantly downregulated (34). The molecular basis of this imbalance involves multiple mechanisms: oxidative stress-activated p38 MAPK signaling promotes the mitochondrial translocation of Drp1, while SIRT1 activity decline due to NAD^+^ depletion weakens the transcriptional regulation of Mfn2 (47). Although the fragmented mitochondrial network appearsmorphologically convenient for EV packaging, its functionality is severely compromised—Drp1-driven excessive mitochondrial fission is intimately linked to cristae remodeling. In young MHCII^+^ donor macrophages, the coordinated actions of OPA1 and the MICOS complex maintain narrow cristae, supporting efficient respiratory chain supercomplex (RCS) assembly and ATP synthesis. It has been clearly established that cristae morphology directly determines the assembly and stability of RCS, thereby governing mitochondrial respiratory efficiency (48, 49). Under aging or metabolic dysregulation, cristae disruption leads to the loss of physical contacts among complexes I–III–IV and a marked impairment of electron transfer efficiency. Consequently, even if fragmented mitochondria retain intact mtDNA-encoded subunits, their capacity for RCS assembly is fundamentally compromised, which explains why an increase in quantity cannot compensate for the decline in quality. At the same time, the OXPHOS collapse (loss of ΔΨm) in aged MHCII^+^ donor macrophages not only reduces ATP synthesis but also causes “quality degradation” of their EV cargo due to mitochondrial depolarization. After being taken up by LCs via the VCAM1-ITGβ1 axis, these depolarized mitochondria not only fail to provide energetic support but may instead trigger inflammatory responses in LCs, thereby converting the donor pathway from “protective” to “pathogenic.” The oxidative stress environment in aged LCs themselves may also lead to the secretion of EVs carrying depolarized mitochondria, which, upon recognition by tMacs, provoke inflammation rather than immunologically silent clearance, constituting the “erroneous response” mechanism of scavenger-side obstruction. Moreover, mitochondrial membrane potential serves as a quantifiable parameter of EV cargo quality and may provide a novel diagnostic marker for assessing donor macrophage function and EV network efficiency (50, 51).

Finally, the age-related downregulation of OPA1 expression (accompanied by sustained fission activation) deprives donor mitochondria of the molecular basis required for fusion competence (49, 52). Even if successfully internalized via the VCAM1-ITGβ1 axis, they cannot form a functional syncytium with the recipient network. In other words, although aged MHCII^+^ macrophages may produce more “packageable” mitochondrial fragments, these fragments are functionally defective products characterized by “increased quantity but degraded quality”. Even if successfully transferred to LCs, they cannot be effectively integrated into the recipient mitochondrial network to exert respiratory function.

#### 3.1.3 OXPHOS system collapse and ATP supply crisis

The combined effects of PGC-1α inhibition and disrupted mitochondrial dynamics ultimately converge on the dysfunction of the oxidative phosphorylation (OXPHOS) system, depriving MHCII^+^ macrophages of their metabolic foundation as “mitochondrial donors”. Seahorse cellular metabolic analyses have shown that the basal oxygen consumption rate (OCR) and ATP-coupled respiration in macrophages from aged Apoe−/− mice are significantly lower than those in young cells, with maximal respiratory capacity and spare respiratory capacity declining by 40–60% (47). Meanwhile, although the testicular interstitium is richly vascularized, age-related thickening of the blood-testis barrier (BTB) and interstitial fibrosis place tMacs in a state of chronic relative hypoxia (53), and testicular macrophages tend to adopt a more inflammatory phenotype with advancing age (53). The chronic activation of hypoxia-inducible factor HIF-1α synergizes with the exhaustion of the CD38-NAD^+^-SIRT3 axis, forcing tMacs under “pseudohypoxic” conditions to undergo metabolic reprogramming from OXPHOS towards aerobic glycolysis (the Warburg effect) (54, 55). However, the glycolytic pathway cannot compensate for the impact of OXPHOS loss on mitochondrial quality—OXPHOS activity is a prerequisite for high membrane potential and low ROS levels in mitochondria, whereas glycolysis-dependent macrophages generally exhibit degenerative changes such as decreased membrane potential, elevated ROS levels, and accumulation of mtDNA damage (48).

One of the core molecular drivers of this OXPHOS collapse is the progressive depletion of NAD^+^. The study by Camacho-Pereira et al. revealed that the expression and activity of the NAD^+^ hydrolase CD38 increase sharply with age, leading to a significant decline in NAD^+^ concentrations across various tissues through its hydrolysis (40). In Cd38-deficient mice, NAD^+^ levels are maintained into advanced age, and mitochondrial respiratory capacity, oxygen consumption rate, and membrane potential remain at levels comparable to those in young mice (56). This protective effect on mitochondrial function depends on the sustained activity of the NAD^+^-dependent deacetylase SIRT3—in the absence of CD38, mitochondrial proteins maintain an appropriate deacetylation state, thereby ensuring the continued operation of oxidative metabolism (56). Conversely, in wild-type aged macrophages, CD38-mediated NAD^+^ depletion restricts SIRT3 activity, leading to hyperacetylation and functional inhibition of respiratory chain complex proteins, with OXPHOS efficiency consequently collapsing.

The loss of SIRT3 activity has particularly profound implications for the supply function of MHCII^+^ macrophages. SIRT3 is the primary deacetylase located in the mitochondrial matrix, and its targets include multiple core OXPHOS components such as respiratory chain complex I (NDUFA9), complex II (SDHA), and ATP synthase (41). In an environment of NAD^+^ depletion caused by high CD38 expression, these key metabolic enzymes remain in a highly acetylated inhibitory state. Even if mtDNA copy number is maintained, the electron transfer efficiency of the respiratory chain is severely impaired. The age-related induction of CD38 expression leads to a progressive decline in NAD^+^ levels, and since NAD^+^ serves as an essential cofactor for the deacetylase activity of SIRT3, its depletion directly limits SIRT3 enzymatic function. The loss of SIRT3 activity consequently renders its downstream targets—including multiple core OXPHOS components such as respiratory chain complexes I and II and ATP synthase—in a highly acetylated and functionally inhibited state, resulting in markedly reduced mitochondrial electron transfer efficiency and slowed oxygen consumption rate. The decline in OXPHOS function not only impairs ATP synthesis capacity but also exacerbates electron leakage from the electron transport chain, triggering substantial production of mitochondria-derived ROS. Notably, accumulated ROS itself acts as a potent inducer of CD38 expression, further upregulating CD38 levels and thereby closing this positive feedback loop, trapping aged macrophages in a self-perpetuating cycle of NAD^+^ depletion and worsening mitochondrial dysfunction (57, 58).

It is also noteworthy that recent research by Vendrov et al. has revealed that age-related increased expression of NOX4 (NADPH oxidase 4) is another critical upstream factor driving mitochondrial dysfunction in macrophages. In aged Apoe Apoe−/− mice, Nox4 deficiency or pharmacological inhibition significantly reduces mitochondrial ROS levels in macrophages, improves mitochondrial respiratory function, and promotes macrophage polarization towards a CD206^+^ anti-inflammatory phenotype (i.e., M2-like state) (48). This finding suggests that the NOX4-ROS axis and the CD38-NAD^+^-SIRT3 axis may constitute a synergistic two-hit mechanism that coordinately regulates mitochondrial quality in aged macrophages.

Collectively, these convergent insults—encompassing PGC-1α suppression, dysregulated mitochondrial dynamics, and OXPHOS collapse—deprive aging MHCII^+^ macrophages of their donor competence, thereby establishing the cellular foundation for the supply-side decompensation of the EV network.

### 3.2 Clearance-side obstruction: impaired recognition and degradative dysfunction in the scavenger subpopulation

Within the EV-mediated mitochondrial outsourcing network, CD206^+^ macrophages, as the dedicated “scavenger” subpopulation, bear the critical responsibility of recognizing and clearing LCs-derived EVs containing defective mitochondria. In the young state, this subpopulation efficiently recognizes EVs through high TREM2 expression, degrades their contents via an intact autophagy-lysosome system, and executes the phagocytic process with a flexible cytoskeleton (28, 29). During aging, however, the scavenger function of CD206^+^ macrophages undergoes systematic deterioration at multiple levels—from receptor recognition to phagosome formation and ultimately lysosomal degradation—with dysfunction manifesting at each step, culminating in “clearance-side obstruction”. This results in prolonged retention of defective mitochondria within LCs and accelerated accumulation of oxidative damage.

#### 3.2.1 Epigenetic silencing and proteolytic shedding of the TREM2 recognition receptor

The recognition of EVs containing defective mitochondria by CD206^+^ macrophages is molecularly based upon high surface expression of the TREM2 receptor. In the aged testicular microenvironment, pro-inflammatory factors—particularly interferon-gamma (IFN-γ)—can induce persistent silencing of scavenger genes such as TREM2 through epigenetic mechanisms. The work of Kang et al. elucidated the molecular basis by which IFN-γ epigenetically represses M2-like genes (e.g., CD206) through an enhancer disassembly mechanism (59). Aging-related chronic low-grade inflammation leads to elevated IFN-γ levels, which may drive the epigenetic silencing of scavenger genes such as TREM2 and CD206 through a similar enhancer disassembly mechanism by analogy with CD206 regulation, thereby depriving CD206^+^ tMacs of their capacity to efficiently recognize and clear LC-derived EVs containing defective mitochondria. On this basis, future studies may further verify whether MAF or its analogous transcription factors indeed directly bind to the TREM2 gene enhancer, and whether IFN-γ inhibits TREM2 transcription by inducing their dissociation—validation of this hypothesis would provide a precise molecular entry point for repairing scavenger function through targeted enhancer remodeling. Although the work of Kang et al. did not directly validate TREM2, subsequent studies have demonstrated that TREM2 is subject to multilayered epigenetic regulation during aging. These mechanisms include DNA methylation, histone modification switches, and non-coding RNAs. Specifically, IFN-γ downregulates TREM2 expression in macrophages through the JNK/NF-κB pathway (60). This impairs their capacity to recognize and clear EVs containing defective mitochondria. Concomitantly, the loss of the inflammation-suppressive function of TREM2 signaling may shift the phagocytic process from immunologically silent clearance to inflammation-amplifying erroneous responses—this constitutes a key molecular basis of scavenger-side obstruction. Furthermore, Huang et al. have revealed that the H3K27ac/H3K27me3 histone modification switch serves as a core mechanism governing TREM2 expression in macrophages—the aging microenvironment progressively replaces H3K27ac with EZH2-mediated H3K27me3 deposition, thereby establishing a transcriptionally silent chromatin state (61). Notably, single-cell sequencing studies in 2025 have further confirmed that TREM2 is a conserved marker of aged testicular macrophages (the Ccl8hi/Cxcl13hi subpopulation), suggesting that it may play a more complex role in age-related scavenger dysfunction (62).

Beyond transcriptional repression, the aged microenvironment can further reduce the level of functional TREM2 receptors on the cell surface by accelerating ectodomain shedding of the TREM2 protein. Studies have shown that TREM2 undergoes sequential proteolytic processing: first, proteases such as ADAM17 mediate ectodomain shedding at the His157-Ser158 site, releasing soluble TREM2 (sTREM2) (63); subsequently, γ-secretase mediates intramembrane cleavage of the transmembrane fragment (CTF), generating an intracellular domain (ICD) that is rapidly degraded (64). Once shed, TREM2 loses its function as an intact membrane receptor, resulting in a reduction in the number of functional receptors on the cell surface. Of particular note, recent research has revealed that aging-related oxidative stress can accelerate TREM2 shedding from the macrophage surface by activating the ROS-NRF2-ADAM17 axis, thereby impairing scavenger function (65). This post-translational mechanism acts synergistically with epigenetic silencing, collectively causing progressive depletion of functional TREM2 receptors on the surface of CD206^+^ macrophages.

#### 3.2.2 Autophagic dysfunction exacerbates the decline of the phagocytic-degradative pathway

Effective scavenger function depends not only upon the recognition and phagocytosis of EVs but also upon the efficient degradation of their contents (especially EVs containing defective mitochondria) following phagosome-lysosome fusion. The autophagy-lysosome pathway occupies a central position in maintaining macrophage phagocytic function. Stranks et al. were the first to demonstrate that autophagy is a key mechanism for maintaining the “youthful state” of macrophages. In mice with hematopoietic system-specific knockout of Atg7, macrophages exhibit a phenotypic spectrum completely consistent with physiological ageing. This spectrum includes impaired phagocytic function, reduced nitrite burst capacity, and increased secretion of pro-inflammatory cytokines. These macrophages also show decreased expression of surface antigen-presenting molecules (MHC II, CD86) and a metabolic shift towards glycolysis.

towards Using ImageStream imaging technology, this study quantitatively demonstrated that autophagic flux (LC3-LysoID colocalization) in bone marrow-derived macrophages from aged (>100-week-old) mice is reduced by more than 50% compared with young (6-week-old) mice, thereby directly establishing a causal link between “declined autophagic flux and macrophage aging” (66).

In the testicular microenvironment, this decline in autophagy is directly linked to the clearance efficiency of the scavenger pathway: the progressive reduction in autophagic flux in aged CD206^+^ tMacs leads to a sustained impairment of their capacity to degrade phagocytosed cargo, including EVs containing defective mitochondria. Incompletely degraded mitochondrial components accumulate within lysosomes, forming a “phagocytic burden” that, in turn, feedback inhibits autophagic-lysosomal function, thereby constituting the core vicious cycle underlying scavenger-side obstruction.

Autophagic dysfunction impairs the efficiency of the scavenger pathway at multiple levels. First, the decline in autophagic flux directly leads to weakened lysosomal acidification capacity and reduced cathepsin activity—in aged rat cells, lysosomal pH rises from the normal ~4.5 to ~6.0, and cathepsin B activity decreases significantly, severely impairing the degradation efficiency of contents after phagosome-lysosome fusion (67). More critically, impaired lysosomal acidification promotes the progressive accumulation of lipofuscin—a mixture of highly oxidized lipids, misfolded proteins, and metal ions that gradually builds up within lysosomes. Studies in microglia by Mendes Lopes et al. have confirmed that lipofuscin-like autofluorescence signals initially accumulate in microglial lysosomes and increase significantly with age (68); in the context of central nervous system aging, the work of Safaiyan et al. has shown that lipofuscin granules are closely associated with myelin debris and occupy substantial physical space within lysosomes (69). Lipofuscin accumulation not only progressively encroaches upon lysosomal space, thereby impairing phagocytic-degradative function, but can also trigger macrophage pyroptosis itself via lysosomal membrane permeabilization (LMP). The released lipofuscin and damage-associated molecular patterns (DAMPs) further activate surrounding tMacs and may be taken up by adjacent LCs, inducing secondary pyroptosis and accelerating the irreversible loss of functional LC reserve. Thus, as a common node connecting aging, lysosomal dysfunction, and pyroptosis, lipofuscin represents a key molecular hub for understanding both the irreversibility of scavenger-side obstruction within the EV network and the progression of LOH (68).

The synergistic effect of reduced lysosomal degradation efficiency and declining phagocytic capacity ultimately leads to the accumulation of incompletely degraded mitochondrial components within CD206^+^ macrophages, forming a “phagocytic burden” that exerts feedback inhibition on their sustained scavenger capacity. Lipofuscin, as a hallmark product of incomplete lysosomal digestion, is a direct manifestation of this process—in cells with an increased phagocytic burden, the further accumulation of lipofuscin occupies more lysosomal volume, exacerbating the vicious cycle.

The situation is further complicated by the fact that aging not only impairs the phagocytic capacity of macrophages towards EVs but also suppresses their broader efferocytosis function—i.e., the clearance of apoptotic cells. A systematic study evaluating the impact of aging on macrophage function demonstrated, through quantitative proteomic analysis, that pathways related to endocytosis, phagocytosis, and efferocytosis are significantly downregulated in aged macrophages compared with young macrophages following lipid loading, and functional experiments further confirmed that their efferocytosis capacity is reduced by approximately 40–50%. The paralysis of efferocytosis denotes a marked decline in the clearance efficiency of apoptotic cells and cellular debris within the testicular microenvironment, including apoptotic LCs, Sertoli cell remnants, and spermatogenic cell debris. This impairment further augments the clearance burden on CD206^+^ macrophages and establishes a vicious cycle wherein apoptotic cell retention fosters damage-associated molecular pattern release, thereby amplifying inflammation and ultimately driving downregulation of efferocytosis receptors (70). Furthermore, senescent cells can suppress macrophage-mediated efferocytosis by upregulating the CD47-QPCT/L axis—senescent cells are not only themselves difficult for macrophages to clear but can also “paralyze” the ability of macrophages to clear bystander apoptotic cells (71). This “bystander effect” may further impair the capacity of CD206^+^ macrophages to clear EVs containing defective mitochondria in the aged testis.

#### 3.2.3 Cytoskeletal rigidity and impaired phagosome formation

Even if EVs are successfully recognized, aged CD206^+^ macrophages face significant obstacles in the execution steps of phagocytic cup formation and phagosome internalization. The underlying cause is primarily the excessive stiffening of the actin cytoskeleton resulting from hyperactivation of the RhoA-ROCK pathway, coupled with impaired filopodia formation mediated by downregulated Rac1 expression.

The study by Saclier et al. revealed a dual role of the RhoA-ROCK1 pathway in macrophage phagocytic function: this pathway normally acts as a molecular brake limiting actin polymerization, and therefore its inhibition—rather than its activation—is a prerequisite for phagocytosis induction and the switch to an anti-inflammatory phenotype (72). During aging, however, oxidative stress and SASP factors persistently induce excessive RhoA activation, leading to excessive crosslinking and increased rigidity of the cytoskeleton, which paradoxically restricts the dynamic remodeling of the phagocytic cup (73). Concurrently, Rac1 expression declines significantly with age, impairing the activation of the Arp2/3 complex and filopodia formation, thereby further weakening the physical capacity of macrophages to envelop EVs (74). Given that EV phagocytosis and bacterial phagocytosis share similar mechanisms of actin cytoskeleton remodeling, it is reasonable to infer that the decline of this Rac1-Arp2/3-filopodia axis likewise impairs the physical envelopment of EVs containing defective mitochondria by aged CD206^+^ macrophages.

Early studies have quantitatively demonstrated that the content of actin, myosin, and vimentin in Kupffer cells from 24-month-old mice is reduced by 32%, 15.1%, and 24.5%, respectively, compared with 6-month-old mice, accompanied by a 56% decrease in phagocytic capacity (75). Furthermore, the age-related degradation of the microtubule network also impairs the transport of phagosomes to the centrosome and their fusion with lysosomes, leading to defective phagosome maturation.

In summary, aged CD206^+^ macrophages suffer full-chain scavenger failure encompassing impaired recognition, defective autophagy–lysosomal degradation, and cytoskeletal rigidity, which prolongs defective mitochondrial retention in LCs. Combined with supply-side collapse, this disrupts the EV network balance and drives LCs below the critical threshold for testosterone synthesis.

### 3.3 Communication decoupling: dual decline in EV quality and recognition efficiency

If supply-side collapse and clearance-side obstruction respectively impair the “input” and “output” functions of the EV-mediated mitochondrial transfer network, then the disruption of communication coupling fundamentally disturbs the efficiency of signal exchange and material transfer between network nodes. As the physical carriers of mitochondrial transfer, EVs are systematically affected by the aging microenvironment throughout their generation, secretion, cargo composition, and recognition and uptake by recipient cells. At the secretory end, the EV generation capacity of aged LCs declines and their cargo undergoes a “toxic transformation”; at the recipient end, the recognition and internalization capacity of LCs for donor EVs also deteriorates. This bidirectional communication impairment leaves LCs trapped in an isolated predicament where they can neither effectively “call for help” nor fully utilize “assistance”, further accelerating the deterioration of the mitochondrial quality crisis.

#### 3.3.1 Decline in ESCRT-dependent EV biogenesis and release, and “toxic transformation” of EV cargo

The EV secretion capacity of aged LCs exhibits a multi-dimensional, progressive decline. Quantitative nanoparticle tracking analysis has shown that the basal EV secretion rate of aged LCs is reduced, and their responsiveness to hCG stimulation is attenuated (25). This secretory impairment is closely related to the functional decline of the MVB system, involving three intertwined molecular mechanisms:

(1) Decline of the ESCRT machinery for MVB biogenesis: The ESCRT-0 component HRS and the ESCRT-I core protein TSG101 are downregulated in aged LCs, impairing the sorting efficiency of ubiquitylated mitochondrial proteins into MVBs (76, 77). This sorting defect not only reduces the generation of intraluminal vesicles (ILVs) containing mitochondrial cargo but also leads to the aggregation of abnormal proteins, triggering MVB trafficking arrest. Similar mechanisms have been observed in neural aging—APOE4 carriers exhibit impaired exosome production, suggesting that ESCRT functional decline may be a cross-tissue feature of aging (76) (2). Increased lysosome-MVB fusion and diversion of the secretory pathway: The frequency of lysosome-MVB fusion increases in aged cells, creating a phenotype of “enhanced endosome-lysosome fusion” that results in more ILVs being degraded rather than released (78). This “degradation bias” represents a compensatory mechanism for the impaired clearance function of aged cells, but at the cost of reduced output of functional EVs. Inhibition of VPS4A (an ESCRT-III-associated ATPase) function further exacerbates MVB diversion towards lysosomes, although this mechanism remains to be experimentally validated in LCs (79) (3). Decreased plasma membrane fluidity and reduced fusion efficiency: The plasma membrane fluidity of aged LCs is significantly reduced, which is closely linked to membrane lipid peroxidation caused by oxidative stress (32). The depletion of glutathione (GSH) and increased oxidative damage in aged cells reduce membrane lipid fluidity, thereby affecting the fusion efficiency mediated by SNARE complexes (such as VAMP7, SNAP23, and STX4) between MVBs and the plasma membrane. Reduced membrane fluidity not only decreases EV release but also alters the membrane lipid composition of EVs, affecting their ability to be recognized by recipient cells (25, 48).

The EV cargo also undergoes a “quality crisis”, with the cargo composition of aged LC-derived EVs undergoing unfavorable changes characterized by “damage signal overload”.

The cargo composition of EVs undergoes a toxic phenotypic switch, transitioning from protective signaling vectors to agents that propagate cellular damage. Operationally, this toxicity is defined by the following molecular signatures (1): elevated mitochondrial damage-associated molecular patterns, including oxidized cardiolipin (ox-CL)—whereas cardiolipin externalization normally functions as an “eat-me” signal for TREM2-mediated recognition, oxidative modification of cardiolipin may impair TREM2 binding affinity or act as a competitive antagonist of scavenger receptors (80) (2); increased leakage of damaged mtDNA into EVs, which contributes to STING-IFN–mediated inflammatory signaling (81) (3); altered mtDNA homeostasis within EVs during aging (82) (4); selective enrichment of pro-inflammatory microRNAs, notably miR-155 (83); and (5) functional deficit of critical antioxidant enzymes such as SOD2, PRDX3, and GPX4 under oxidative stress, as evidenced by the restorative capacity of exogenous EVs (84).

Notably, miR-145-5p derived from Sertoli cells can be transferred to LCs via exosomes to inhibit steroidogenesis, suggesting an imbalance in EV-mediated paracrine regulation within the aging microenvironment (85). These alterations mean that aged LCs not only output fewer EVs but also that the EVs they do output are of “inferior quality”—they carry oxidatively damaged proteins, mutant mtDNA, and pro-inflammatory signals, potentially transmitting “damage alarms” rather than “clearance requests” to tMacs. Under normal circumstances, EVs containing damaged mitochondria are recognized by TREM2 on tMacs via exposed cardiolipin, triggering phagocytic clearance. However, oxidatively damaged cardiolipin in aged EVs may alter TREM2 recognition affinity or competitively inhibit scavenger receptors, leading tMacs to “reject” them or mount an “erroneous response” (inflammatory activation rather than phagocytosis).

#### 3.3.2 Decline in receptor recognition and internalization capacity

In addition to the functional decline of both donor and recipient cells, aging also disrupts the communication coupling between them through three mechanisms: downregulation of recognition receptors, impairment of the endocytic machinery, and the establishment of physical barriers within the microenvironment. The expression of vascular cell adhesion molecule 1 (VCAM1) on the surface of aged LCs declines significantly with age, directly impairing their recognition and uptake efficiency of EVs derived from MHCII^+^ macrophages (38, 41). Single-cell transcriptomic analysis has further confirmed that in aged testes (60–69 years), the strength and connectivity of the Vcam signaling pathway (involving Vcam1-Itga4/Itgb1 interactions) are markedly reduced (38). Age-related integrin dysfunction further exacerbates the impairment of EV uptake. The internalization of EVs mediated by the integrin–ITGβ1–VCAM1 axis depends on clathrin-mediated endocytosis (CME) and dynamin-dependent membrane scission. In aged cells, declining levels of the βPIX–GIT complex lead to reduced CME efficiency, thereby impairing integrin internalization. Concurrently, disrupted interaction between the clathrin adaptor protein AP2 and the integrin α-chain weakens CME efficiency (86, 87). Defective BRAG2/GEP100/IQSec1-mediated Arf5 activation further compromises this process. Together, these defects result in a marked decline in the uptake of healthy mitochondria-containing EVs derived from MHCII^+^ donor tMacs by LCs. Notably, the integrity of RhoA signaling is critical for macrophage endocytosis and phagocytosis, and macrophages in different polarization states exhibit differential responses to RhoA pathway interference, suggesting that age-related dysregulation of RhoA signaling may further impair the effective uptake of EVs by LCs (88).

Finally, age-related extracellular matrix (ECM) remodeling may alter the diffusion kinetics of EVs within the interstitium, further hindering efficient mitochondrial transfer. The aged testicular interstitium undergoes significant ECM remodeling: thickening of the basement membrane and increased deposition of fibronectin and laminin collectively form a dense ECM barrier (89, 90). These changes may reduce tissue porosity, impede the penetration of EVs with diameters of 100–150 nm, and potentially “trap” EVs, causing them to remain near secretory cells and fail to reach distant tMacs, thereby creating “information islands”. The dTECM (decellularized testicular extracellular matrix) study by Chi et al. provides proof-of-concept for reversing this coupling disruption: remodeling the aged microenvironment with young dTECM not only restored the steroidogenic function of LCs but also significantly increased the number of CD206^+^ anti-inflammatory M2 macrophages in the testicular interstitium and alleviated age-related chronic inflammation (91). This confirms that the ECM microenvironment is a key modifiable factor regulating LCs-tMacs communication coupling.

## 4 The cascade mechanisms by which EV network dysregulation drives LOH

**Figure 3:**
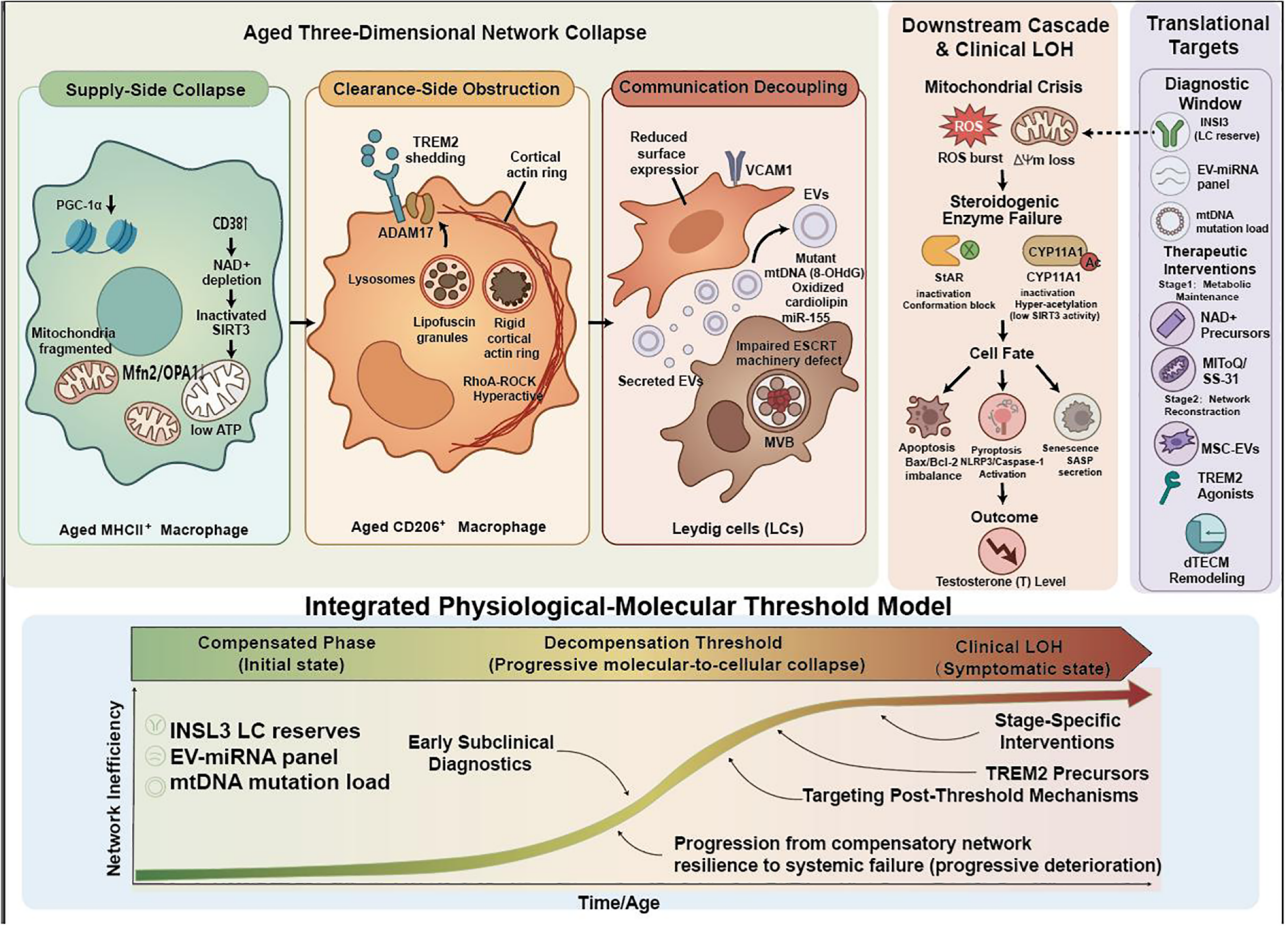
The decompensation threshold model. Schematic illustration of the phase transition from compensated aging to clinical LOH.

In the face of the aforementioned three-dimensional breakdown, the EV-mediated mitochondrial outsourcing network does not collapse immediately. During early aging, LCs can transiently maintain stable testosterone synthesis through compensatory mechanisms such as upregulating PGC-1α to enhance mitochondrial biogenesis, promoting the fusion of healthy mitochondria, and relying on feedback LH stimulation via the hypothalamic-pituitary-gonadal (HPG) axis. However, these compensatory mechanisms have inherent limits. When the clearance rate of the scavenger pathway or the replenishment rate of the donor pathway falls below the rate of mitochondrial damage generation, the network renewal rate will drop below the critical threshold required to sustain LC function—the “decompensation threshold”—and the system will undergo a phase transition from compensation to decompensation, at which point clinical LOH manifests. This threshold model not only explains why only a subset of aging men develop LOH (due to individual differences in network reserve) and why symptoms progressively worsen (the self-amplifying cycle triggered by ROS burst after crossing the threshold) but also reveals why testosterone supplementation alone has limited efficacy (because it fails to repair the three-dimensional damage to the network itself). The concept of the decompensation threshold and the phase transition from compensated aging to clinical LOH is illustrated in **Figure 3**. The following sections will sequentially dissect the cascade events that drive LOH progression once the threshold has been crossed.

### 4.1 Network imbalance leads to LC dysfunction

The immediate consequence of declining network efficiency is the progressive deterioration of mitochondrial quality within LCs. Network imbalance precipitates excessive reactive oxygen species (ROS) generation. ROS-induced damage to mitochondrial DNA and respiratory chain complexes further compromises electron transfer efficiency, thereby establishing a self-amplifying vicious cycle in which ROS accumulation promotes cellular damage, which in turn exacerbates ROS production (92). On the one hand, ROS activates p38 MAPK, thereby inhibiting CREB and subsequently downregulating steroidogenic acute regulatory protein (StAR). On the other hand, ROS induces cyclooxygenase-2 (COX-2) overexpression, and the resultant downstream inflammatory mediators further suppress steroidogenic enzyme activity (92, 93). Additionally, ROS drives cellular senescence via the p53/p21 pathway (94). Ultimately, LCs undergo an irreversible transition from an early stage of metabolic adaptation, sustained by compensatory PGC-1α activity and antioxidant enzyme function, to a stage of metabolic crisis characterized by the sequential progression of ROS accumulation, cumulative damage, and cell death. This transition arises from the overwhelming failure of the mitochondrial quality control system and is accompanied by the complete collapse of testosterone biosynthetic capacity.

The oxidative stress environment triggered by the mitochondrial quality crisis inflicts a devastating blow on the steroidogenic enzyme system of LCs. The rate-limiting steps of testosterone synthesis—the transport of cholesterol from the outer to the inner mitochondrial membrane and subsequent side-chain cleavage—are highly dependent on mitochondrial membrane potential, intact cristae structure, and a precise redox environment. The collapse of mitochondrial membrane potential and the ROS burst caused by network dysregulation impair this delicate process at multiple levels.

First, to exert its cholesterol transport function on the outer mitochondrial membrane (OMM), StAR must undergo a pH-dependent molten globule conformational change (transitioning from a tightly folded state to a partially unfolded state), a transformation that depends on the proton gradient maintained by the mitochondrial membrane potential (ΔΨm). The collapse of the proton gradient due to loss of membrane potential prevents StAR from completing this essential conformational “switch”, causing StAR to “stall” on the membrane and lose its ability to continuously transport cholesterol (95, 96).

Second, CYP11A1 and other CYP450 family steroidogenic enzymes are inactivated by oxidative modifications. The study by Li et al. revealed that 1-nitropyrene (1-NP) exposure increases the acetylation levels of mitochondrial steroidogenic enzymes (including StAR, CYP11A1, and 3β-HSD1) by inhibiting SIRT3 activity, leading to decreased protein stability and loss of catalytic activity (97).

Third, the structural disruption of mitochondria-associated endoplasmic reticulum membranes (MAMs) further blocks the spatial organization of steroidogenesis. Steroidogenesis depends on close physical contact and efficient metabolite transfer between mitochondria and the endoplasmic reticulum—after cholesterol is converted to pregnenolone within mitochondria, it must be rapidly transported to the endoplasmic reticulum for further processing by enzymes such as 3β-HSD1 and CYP17A1. Mitochondrial fragmentation and imbalanced network dynamics disrupt the integrity of MAMs, leading to reduced transport efficiency of intermediate metabolites and further impairment of testosterone synthesis capacity (98).

Taken as a whole, the synergistic effects of ROS accumulation and SIRT3 inactivation drive LCs into a comprehensive decline in steroidogenic function. A recent review on testicular aging has explicitly stated that mitochondrial dysfunction is a key driver of testicular aging—the decline in mitochondrial membrane potential directly impairs testosterone synthesis efficiency, whereas ROS accumulation accelerates cellular senescence programs (99). Notably, damage to steroidogenic enzymes often exhibits functional irreversibility—CYP450 enzymes generate ROS during catalysis, and their active centers are exquisitely sensitive to oxidative stress. Once oxidative damage occurs, their enzymatic activity is difficult to fully restore through simple antioxidant therapy (100). This constitutes an important molecular basis for the irreversible progression of LOH driven by network dysregulation.

### 4.2 Reduction in cell number

Network dysregulation not only impairs the function of individual LCs but also leads to the irreversible reduction in the number of functional LCs through multiple intertwined cell death pathways. As terminally differentiated cells, adult LCs have extremely limited proliferative and renewal capacity—although a pool of stem LCs (SLCs) can differentiate into new LCs, this regenerative capacity declines significantly with age (101). Therefore, the death of each functional LC represents a permanent loss in the total capacity for testosterone synthesis. Network dysregulation induces LC death through the following mutually amplifying death pathways:

(1) Apoptosis. Insufficient ATP supply and oxidative stress jointly activate the mitochondrial apoptotic pathway. The collapse of mitochondrial membrane potential leads to the release of cytochrome c from the intermembrane space into the cytosol. There, it forms the apoptosome with Apaf-1 and procaspase-9. This event activates the caspase cascade and ultimately leads to DNA fragmentation and cell disintegration. Studies have shown that the Bcl-2/Bax ratio is significantly reduced in aged LCs, indicating increased susceptibility to apoptosis (102) (2). Pyroptosis. Chronic inflammation in the aged testicular microenvironment induces LC pyroptosis through inflammasome activation. Ruan et al. found that exposure to environmental toxicants can induce caspase-1/GSDMD-dependent pyroptosis in LCs by activating the NLRP3 inflammasome, leading to the formation of cell membrane pores, release of cellular contents, and amplification of local inflammation (103). The DAMPs (such as mtDNA and cardiolipin) released during pyroptosis further activate tMacs, creating a vicious cycle of “cell death-inflammation-more cell death” (3). Senescence. Persistent oxidative stress and DNA damage push LCs into a state of cellular senescence. Senescent LCs not only lose their proliferative capacity and normal function but also secrete pro-inflammatory factors such as IL-6 and TNF-α through the SASP, disrupting the microenvironment of neighboring cells (104). Li et al. demonstrated that premature senescence of LCs initiated by mitochondrial dysfunction partially participates in the downregulation of steroidogenic enzymes induced by 1-NP. MitoQ treatment can prevent 1-NP-induced LC senescence, suggesting that mitochondria-targeted interventions may have dual effects in preserving both the number and function of LCs (97).

Collectively, the three death pathways—apoptosis, pyroptosis, and senescence—do not operate independently but are intertwined and mutually amplifying. When the number of functional LCs falls below a certain critical threshold, the compensatory overwork of the remaining cells accelerates their functional decline, ultimately leading to a precipitous drop in total testosterone synthesis capacity. This constitutes the cytological basis for the rapid exacerbation of LOH symptoms in advanced stages.

### 4.3 Disruption of the paracrine network

The functional decline of LCs not only affects testosterone synthesis itself but also disrupts the homeostasis of the entire spermatogenic microenvironment through remodeling of paracrine signaling, creating a cascade effect that impacts spermatogenesis and male fertility.

Testosterone, as a key paracrine factor supporting spermatogenesis, exerts a direct impact on Sertoli cell function and the integrity of the blood-testis barrier (BTB) through its declining interstitial concentration. Sertoli cells are the only somatic cells within the seminiferous epithelium that directly contact developing germ cells, and their function is highly dependent on testosterone signals derived from LCs (105). Testosterone, by binding to androgen receptors (AR) within Sertoli cells, regulates the expression of a series of genes crucial for spermatogenesis (85). Under LOH conditions, testosterone deficiency leads to the functional deterioration of Sertoli cells and compromised BTB integrity, subsequently causing meiotic arrest and impaired sperm release (106, 107).

In addition to testosterone, a variety of paracrine factors secreted by LCs are essential for spermatogenesis. Activin A, a key factor secreted by both fetal and adult LCs, promotes Sertoli cell proliferation and the formation of seminiferous cords (108). Furthermore, LCs regulate Sertoli cell function through the secretion of testosterone and paracrine signals (such as PDGF and DHH), while Sertoli cells in turn secrete growth factors such as GDNF, FGF2, and IGF1 to maintain the spermatogonial stem cell (SSC) niche (18, 109, 110). LC dysfunction caused by network dysregulation indirectly impairs the self-renewal and differentiation capacity of SSCs by disrupting this “LCs-Sertoli-germ cell” paracrine axis, further exacerbating the deterioration of the spermatogenic microenvironment.

Importantly, EVs themselves are key mediators of intercellular communication within the testicular spermatogenic microenvironment. Zheng et al. (2025) discovered that EV-mediated transfer of let-7b/7c promotes the proliferation of transitional spermatogonia in neonatal mouse testes (111). The “toxic transformation” of EV cargo during aging implies that LCs not only reduce normal support signals to Sertoli cells but may also disseminate “damage signals” throughout the spermatogenic microenvironment by releasing EVs enriched in pro-inflammatory miRNAs and oxidatively damaged proteins, amplifying local inflammation and tissue injury. The review by Hofmann and McBeath further emphasized that paracrine and juxtacrine communication between Sertoli cells and germ cells is central to maintaining the SSC niche (112). Therefore, LC network dysregulation indirectly undermines the supportive capacity of Sertoli cells towards SSCs by altering EV-mediated signal transmission.

In summary, LC dysfunction driven by network dysregulation transmits damage from the interstitium to the seminiferous tubules through three pathways—testosterone deficiency, aberrant paracrine factors, and the “toxic transformation” of EV signals—ultimately leading to impaired spermatogenesis and reduced male fertility. The disruption of this paracrine network further explains why exogenous testosterone supplementation alone cannot fully restore fertility in LOH patients—although testosterone replacement therapy (TRT) can partially compensate for testosterone deficiency, it cannot replace the complex regulatory role of LC-derived EVs in maintaining Sertoli cell-germ cell communication.

## 5 Clinical translation: the EV network as a diagnostic and therapeutic target for LOH

The conceptualization of the EV-mediated mitochondrial outsourcing network opens a new paradigm for the clinical management of LOH, shifting the focus from “symptom relief” towards “network repair”. Based on the network decompensation threshold model and cascade mechanisms elaborated above, this section explores the prospects for clinical translation from three perspectives: diagnostic biomarkers, therapeutic strategies, and preventive interventions.

### 5.1 Diagnostic window: identifying at-risk individuals during the compensated phase

The current clinical diagnosis of LOH relies primarily on the measurement of serum total testosterone levels, supplemented by the calculation of free testosterone indices using sex hormone-binding globulin (SHBG). However, serum testosterone levels decline significantly only after network efficiency has fallen below the decompensation threshold and LC function has undergone substantial deterioration—this means that traditional diagnostic methods capture the endpoint events of the “decompensated phase” rather than the early warning signals of the “compensated phase”. Developing biomarkers capable of identifying the risk of declining EV network efficiency during the compensated phase is crucial for early intervention in LOH.

INSL3 is a direct indicator of LC functional reserve. Insulin-like factor 3 (INSL3) is a peptide hormone constitutively secreted by mature LCs, and its expression level is closely correlated with the differentiation status and functional reserve of LCs. Unlike testosterone, INSL3 secretion is not acutely regulated by the HPG axis; instead, it directly reflects the number and differentiation maturity of LCs (113). Studies have shown that INSL3 is significantly more sensitive to LC damage than testosterone—in adult men, a decline in INSL3 levels can be detected several years before a decrease in testosterone, signaling testicular endocrine dysfunction (114). A multi-omics study on testicular aging has also confirmed that the production of both testosterone and INSL3 by aged LCs declines simultaneously, reflecting a dual impairment of testicular function (53). It must be noted that evidence linking TREM2 signaling to INSL3 regulation currently derives from cryptorchidism studies rather than from aging or late-onset hypogonadism (LOH) models; specifically, Trem2 knockout mice exhibit reduced LC numbers and significantly decreased INSL3 levels (115). Consequently, establishing INSL3 as a biomarker reflective of EV outsourcing network functional status requires prospective validation of its diagnostic performance in aging cohorts. Nevertheless, serum INSL3 detection remains a promising preferred candidate biomarker for assessing network efficiency and identifying high-risk individuals during the compensated phase.

In addition to INSL3, serum EV-miRNA profiles (such as the let-7b/7c ratio and levels of miR-155 and miR-146a) and mtDNA mutation burden together constitute a multidimensional biomarker system for evaluating EV network function. Zheng et al. demonstrated that testicular EVs regulate spermatogonial proliferation through let-7b/7c transfer, suggesting that changes in EV-miRNA content can reflect the secretory activity of testicular interstitial cells (111). By combining serum INSL3 (reflecting LC functional reserve) with EV-miRNA profiles (reflecting intercellular communication efficiency), a composite scoring model for predicting LOH risk could be constructed, enabling the early identification of individuals in the compensated phase (111, 116).

### 5.2 Therapeutic strategies: interventions targeting network repair

Based on the three-dimensional breakdown mechanisms of the EV outsourcing network, therapeutic strategies should shift from simply supplementing testosterone towards multi-level, multi-target network repair (**Table 1**).

**Table 1:** Therapeutic strategies for leydig cell function restoration.

Therapeutic target	Intervention/approach	Proposed mechanism of action	Key evidence	Evidence level	Human validation status	Ref.
Network repair: scavenger-side	TREM2 agonists (monoclonal antibodies or small-molecule activators)	Enhance CD206^+i^ tMac recognition and phagocytic clearance of LC-derived EVs containing defective mitochondria; restore anti-inflammatory microenvironment via TREM2-DAP12-Syk-PI3K-Akt signaling	*Trem2* KO mice show 35% testosterone reduction with defective mitochondrial accumulation in LCs; TREM2 shedding by ADAM17 and epigenetic silencing via IFN-γ contribute to age-related scavenger dysfunction	Preclinical (genetic and pharmacological models)	Not validated; BTB penetration poses a major obstacle for antibody-based agonists	(12, 13) (43–49)
Network repair: donor-side	PGC-1α activators (ZLN005, exercise mimetics)	Reactivate mitochondrial biogenesis in MHCII^+^ tMacs via AMPK-PGC-1α-NRF1/2 axis; restore fusion-fission balance (Mfn2/OPA1 upregulation, Drp1 normalization)	Age-related PGC-1α suppression via NF-κB and p53 leads to mtDNA copy-number reduction (30–50%) and OXPHOS collapse; ZLN005 rescues mitochondrial function in aged macrophages	Preclinical (cellular and animal models)	Not validated for testicular application	(20, 26–30)
Network repair: communication coupling	VCAM1-ITGβ1/α4 axis enhancers (VCAM1 overexpression vectors or integrin-activating peptides)	Restore LC recognition and uptake of donor EVs from MHCII^hi^ tMacs; improve dynamin-dependent endocytosis and mitochondrial integration into LC network	LC-specific *Vcam1* KO reduces EV uptake by >70%, lowers ATP by ~40%, and decreases testosterone by 45–50%; scRNA-seq confirms VCAM1-ITGA4/ITGB1 pathway weakening in aged human testes	Preclinical (genetic KO models)	Indirect support only (human scRNA-seq correlational data)	(12, 23, 25, 64, 66, 67)
Network repair: multi-modal cell therapy	MSC-derived small extracellular vesicles (MSC-sEVs)	Deliver functional mitochondria directly to LCs, bypassing the compromised donor pathway; deliver anti-inflammatory and pro-survival cargoes (VEGF, miRNAs) to tMacs and LCs	MSC-sEVs restore steroidogenic enzyme expression in damaged LCs; VEGF concentration in secretome correlates with testosterone-stimulatory potency; MSC-sEVs carry intact,respiration-competent mitochondria	Preclinical (*in vitro* and *in vivo* injury models)	Phase I/II trials ongoing for non-testicular indications; no LOH-specific human data	(94–96)
Network repair: microenvironment remodeling	Young decellularized testicular ECM (dTECM) hydrogels	Restore supportive interstitial architecture; reduce ECM stiffness to normalize PIEZO1-mediated Ca^2+^-ROS signaling; promote anti-inflammatory M2 tMac polarization	Young dTECM rescues steroidogenic differentiation and testosterone production in aged LCs; increases CD206^+^ M2 macrophage abundance in aged testes	Preclinical (animal models)	Not validated	(71)
Mitochondrial protection: antioxidant defence	Mitochondria-targeted antioxidants (MitoQ, SS-31)	Scavenge mtROS at the source (matrix-targeted MitoQ; cardiolipin-binding SS-31); protect respiratory chain complexes from oxidative damage; prevent StAR and CYP11A1 dysfunction	MitoQ prevents 1-NP-induced LC premature senescence and restores steroidogenic enzyme expression; SS-31 preserves mitochondrial cristae integrity in aging models	Preclinical (animal and cell models)	MitoQ: Phase II trials completed for other indications (kidney disease, multiple sclerosis); no LOH trials	(75)
Metabolic rescue: NAD^+^ restoration	NAD^+^ precursors (NMN, NR)	Pleiotropic, cell-type-specific metabolic repair: (i) in donor tMacs—restore SIRT1-PGC-1α axis to reactivate mitochondrial biogenesis; (ii) in LCs—SIRT3-mediated deacetylation maintains StAR, CYP11A1 and 3β-HSD1 stability; (iii) in scavenger tMacs—SIRT1-TFEB axis enhances autophagic-lysosomal clearance	NMN increases testicular NAD^+^ and serum testosterone, and protects LCs and germ cells in ischemia-reperfusion injury; NR raises NAD^+^ and improves mitochondrial respiration in aged macrophages; SIRT3 OE rescues steroidogenic enzyme acetylation	Preclinical (animal models); Phase I–II human trials for NMN/NR (anti-aging, metabolic syndrome)	NMN/NR: multiple human trials show safety and peripheral metabolic benefits; no testis-specific PK or efficacy data published	(40–42, 97–99)
Metabolic rescue: lifestyle intervention	Caloric restriction/intermittent fasting; regular aerobic and resistance exercise	Activate AMPK-SIRT1-PGC-1α axis; reduce systemic inflammaging; improve macrophage mitochondrial metabolism and polarization balance	Caloric restriction attenuates oxidative and inflammatory markers in aged rat testis; exercise restores PGC-1α responsiveness in aged macrophages	Preclinical (animal models); extensive human cohort studies for general metabolic health	Exercise and caloric restriction widely recommended for metabolic health; no LOH-specific RCTs with testosterone as primary endpoint	(24, 28)
HPG-axis modulation	hCG stimulation	Directly activate LC LH receptors to stimulate endogenous testosterone synthesis; potential partial restoration of donor-side PGC-1α via cAMP signaling	hCG is clinically used for male hypogonadism and fertility preservation; may upregulate PGC-1α (hypothesis, no direct evidence)	Clinical (established practice for hypogonadotropic hypogonadism and fertility)	Widely used; however, network-repair effect on EV outsourcing remains speculative	(105)
HPG-axis modulation	Selective estrogen receptor modulators (SERMs; clomiphene, enclomiphene)	Antagonise hypothalamic-pituitary estrogen negative feedback → increase endogenous LH and FSH → stimulate LC function; *untested hypothesis*: direct NF-κB inhibition in tMacs may promote M2 anti-inflammatory polarization	Polari et al. demonstrated SERMs inhibit NF-κB and promote M2 phenotype in human CD14^+^ monocytes (not tMacs); clomiphene increases testosterone in hypogonadal men	Clinical (off-label use for male hypogonadism and fertility); preclinical (monocyte data only)	SERMs used clinically for male infertility; translation to tMac EV-network modulation is an *untested hypothesis* requiring testis-specific validation	(101, 105)
Symptom management	Testosterone replacement therapy (TRT; gels, injections, patches)	Restore circulating testosterone levels to alleviate LOH symptoms (fatigue, reduced libido, muscle loss, cognitive decline)	Meta-analyses and registry studies confirm symptomatic benefit; TRT suppresses endogenous LH via negative feedback, potentially further compromising residual LC function and EV-network compensatory capacity	Clinical (FDA-approved for male hypogonadism); long-term safety data from registry studies	Approved and widely prescribed; does *not* repair the underlying EV-network pathology; concerns regarding fertility suppression and cardiovascular risk remain debated	(104, 105)
Novel delivery: EV engineering	Engineered LC-targeted EVs (loaded with healthy mitochondria, SIRT3 activators, or antioxidant enzymes)	Surface modification with VCAM1-ITGβ1/α4 ligands for targeted LC uptake; internal cargo replaces defective mitochondria or rescues SIRT3 activity	Sertoli cell-derived sEVs cross BTB and deliver functional cargoes to germ cells; conceptually extendable to LC-targeted delivery	Proof-of-concept only	Not validated; manufacturing standardization, batch consistency, and scalability remain critical bottlenecks	(102)
Novel delivery: BTB modulation	Ultrasound-microbubble-assisted BTB opening; cytokine-mediated transient tight-junction modulation	Create a therapeutic window for systemic macromolecules (antibodies, EVs) to access testicular interstitium; reversible approach minimizing germ-epithelium damage	Demonstrated in brain-tumour models for blood-brain barrier opening; application to BTB remains speculative	Conceptual only	Not validated; safety regarding germinal epithelium integrity is a major concern	—

#### 5.2.1 MSC-derived small extracellular vesicles: a cell-free regenerative strategy

Among the therapeutic strategies outlined above, MSC-sEVs are rapidly emerging as a research focus in the field of male reproductive repair owing to their unique biological advantages. Compared with direct MSC transplantation, MSC-sEVs offer low immunogenicity, no tumorigenic risk, the ability to cross biological barriers, and ease of standardized production and storage. A recent systematic review has summarized the therapeutic effects of adipose-, bone marrow-, and umbilical cord-derived MSC-exosomes in preclinical models of male reproductive disorders; available evidence indicates that MSC-exosomes promote the restoration of spermatogenesis and reestablish hormonal balance through multiple mechanisms, including attenuating oxidative stress, suppressing inflammatory responses, improving vascular function, and repairing damaged testicular architecture (117). The feasibility of large-scale standardized production is further supported by studies demonstrating that immortalized human MSC secretomes retain biological potency and lack transforming activity (118). With respect to the protection of LC function, the MSC secretome stimulates testosterone secretion in primary LC in a dose-dependent manner, an effect positively correlated with VEGF concentration that has been validated as a surrogate potency marker (119). Extending this concept to the EV-mediated mitochondrial outsourcing network, MSC-sEVs can not only modulate the polarization state of tMacs through their cargo of bioactive molecules, but more importantly, MSC-sEVs themselves are enriched with functionally intact mitochondria and may thus serve as a direct source of “exogenous healthy mitochondria.” These mitochondria can be taken up by LCs via the VCAM1-ITGβ1 axis or similar recognition mechanisms, thereby bypassing the declining MHCII^+^ donor pathway and directly replenishing the mitochondrial pool of LCs.

#### 5.2.2 NAD^+^ precursors and sirtuin activation: metabolic rescue of the network

NAD^+^ precursor supplementation represents another highly promising network repair strategy, whose mechanism of action closely aligns with the core mechanism of donor-side collapse—namely, the dysregulation of the CD38–NAD^+^–SIRT3 axis in MHCII^+^ macrophages. Adequate NAD^+^ levels support the activities of nuclear SIRT1 and SIRT6, as well as mitochondrial SIRT3. SIRT1 regulates spermatogonial stem cell self-renewal, meiotic fidelity, blood-testis barrier integrity, and testosterone synthesis. SIRT3 acts as a mitochondrial sentinel. It enhances antioxidant defense by activating SOD2, supports oxidative phosphorylation, and facilitates steroidogenesis. SIRT6 maintains genomic and telomeric stability through histone deacetylation (120).defense

Direct experimental evidence further supports the translational potential of NAD^+^ precursors in testicular protection. Studies have demonstrated that NMN supplementation significantly elevates testicular NAD^+^ content, increases serum testosterone levels, prevents LC and germ cell damage, and improves sperm counts, with the underlying mechanism involving remodeling of the inflammatory properties of macrophages and neutrophils to modulate the immune microenvironment (121, 122). Importantly, these studies confirmed that NMN supplementation produced no observable adverse effects in mouse models, providing preliminary safety evidence for its clinical translation.

Within the network repair framework constructed in this review, NAD^+^ precursor (NMN/NR) supplementation can achieve a “three-in-one” therapeutic effect. First, in MHCII^+^ donor macrophages, restoration of SIRT1 activity leads to PGC-1α deacetylation. This reestablishes mitochondrial biogenesis and restores the supply of “healthy mitochondria” (repairing the donor side). Second, in LCs, SIRT3-mediated deacetylation maintains the protein stability and catalytic activity of steroidogenic enzymes such as StAR, CYP11A1, and 3β-HSD1 (preserving LC function) (97). Third, in CD206^+^ scavenger macrophages, restoration of the SIRT1--TFEB axis enhances autophagic-lysosomal function. This improves the degradation efficiency of EVs containing defective mitochondria (alleviating scavenger-side obstruction). This “three-in-one” effect provides a theoretical basis for NAD^+^ precursors as a core component of a “metabolic prescription” for LOH.

Notably, certain existing endocrine therapeutic modalities may exert network-repair effects that have hitherto been underappreciated. hCG therapy—a classical clinical regimen for stimulating endogenous testosterone secretion—not only promotes steroidogenesis by activating LH receptors on LCs, but may also partially restore donor-side function through PGC-1α upregulation. Nevertheless, this mechanism remains a prospective hypothesis currently lacking direct experimental validation. Selective estrogen receptor modulators (SERMs), such as clomiphene, elevate endogenous LH and FSH levels by antagonizing hypothalamic–pituitary estrogen negative feedback, thereby indirectly stimulating LC function (123). Polari et al. first demonstrated in human CD14^+^ monocytes—notably, not testicular macrophages—that multiple SERMs can directly promote an M2 anti-inflammatory phenotype by inhibiting the NF-κB pathway (124). On this basis, a testable scientific hypothesis can be advanced: SERMs may indirectly improve scavenger-side EV network function by modulating testicular macrophage polarization. However, given that this study did not involve tMacs or the testicular microenvironment, and that the SERM types examined differ from clomiphene—the agent commonly used in clinical practice for male infertility—this hypothesis remains entirely untested. It should not be regarded as an established mechanism until direct validation is obtained in testis-specific experimental models. Any inferential effect of SERMs on testicular macrophage polarization constitutes a speculative inference (124).

Given that the “three-dimensional decompensation” of the EV network involves the synergistic collapse of the donor side, scavenger side, and intercellular communication, single-target interventions are unlikely to suffice for network restoration. Combinatorial strategies—for example, NAD^+^ precursors (repairing donor-side metabolism), TREM2 agonists (enhancing scavenger recognition), and young dTECM hydrogels (remodeling the communication microenvironment)—are, at the theoretical level, conceptual frameworks rather than validated therapeutic regimens. An analogous concept—namely, achieving multi-target synergy by correcting metabolic reprogramming and mitochondrial function—has already received preliminary validation in the fields of type 2 diabetes and cardiovascular disease, and its applicability in LOH warrants systematic evaluation in preclinical models. Furthermore, a combined strategy of testosterone replacement therapy plus NAD^+^ precursor supplementation may theoretically achieve the dual benefit of symptomatic relief concomitant with network repair, yet this combination likewise requires experimental validation.

#### 5.2.3 EV-mediated targeted delivery: crossing the blood–testis barrier

A critical obstacle facing network repair therapies is the blood–testis barrier (BTB). While the BTB safeguards the spermatogenic microenvironment from exogenous harmful agents, it also restricts the access of most therapeutic drugs to the testicular interstitium and seminiferous tubules. EVs themselves—particularly those derived from the testicular microenvironment—possess an inherent capacity to traverse the BTB. A breakthrough study by Chen et al. demonstrated that Sertoli cell-derived small extracellular vesicles (SC-sEVs) are capable of crossing the BTB and entering germ cells. The authors further successfully loaded an miR-24-3p inhibitor into SC-sEVs, creating the nanotherapeutic SC-sEV@miR-24-3p inhibitor, which significantly improved sperm motility, *in vitro* fertilization success rates, blastocyst formation rates, and litter size in a gossypol-induced asthenozoospermia mouse model (125). The significance of this proof-of-concept study extends well beyond the treatment of asthenozoospermia—it represents the first systematic demonstration of the feasibility of employing testicular microenvironment-derived EVs as inherent carriers for delivery across the BTB. Extending this strategy to the network repair framework, one can envision the design of engineered, LC-targeted EVs whose surfaces are modified with ligands to enhance VCAM1–ITGβ1 recognition efficiency, and whose cargoes are loaded with healthy mitochondria, antioxidant enzymes, or SIRT3 activators, thereby enabling the precise “material delivery” to LCs.

The BTB challenge is critically size-dependent across therapeutic modalities (1). NAD^+^ precursors (NMN/NR). As small molecules (~300–400 Da), they may partially cross the BTB after systemic administration based on physicochemical principles; however, direct pharmacokinetic data measuring interstitial NAD^+^ elevation in the testis are currently absent, and peripheral increases observed in liver and muscle cannot be extrapolated to the testicular interstitium (126) (2). TREM2 agonists. Whether monoclonal antibodies or peptide agonists, their large molecular weight (>10 kDa) and Fc-mediated clearance render effective BTB traversal virtually impossible—as demonstrated for the structurally analogous blood–brain barrier, where unmodified TREM2 antibodies require engineered transport vehicles (e.g., TfR-mediated transcytosis) to achieve parenchymal penetration (127, 128). Following systemic dosing, interstitial drug concentrations may reach <1–5% of peripheral blood levels, insufficient to activate CD206^+^ testicular macrophages (3). MSC-EVs and exogenous mitochondria. Native MSC-EVs possess limited intrinsic BTB-crossing capacity. Although engineered umbilical cord MSC-EVs have been demonstrated to traverse the BTB when functionalized with targeting ligands (129), and Sertoli cell-derived sEVs possess intrinsic BTB-crossing capacity (125, 130), allogeneic MSC-EVs lack Sertoli-specific homing signals, resulting in negligible testicular targeting efficiency. Intravenous infusion risks >90% hepatic and splenic sequestration.

Consequently, systemic administration alone is likely inadequate for macromolecular biologics and EVs. Clinical realism demands the development of local or semi-local delivery strategies: (i) Intratesticular microinjection—direct delivery of MSC-EVs, TREM2 agonists, or drug-loaded nanoparticles into the testicular interstitium bypasses the BTB entirely, as demonstrated by lipid-nanoparticle-mediated spermatogenesis restoration (131) and cationic lipid-fibroin complex delivery (132); (ii) Sustained-release depots—young decellularized testicular ECM (dTECM) hydrogels or PLGA microparticles implanted locally could serve as interstitial reservoirs, continuously releasing NAD^+^ precursors or small-molecule TREM2 agonists while balancing targeting precision with reduced invasiveness; (iii) Reversible BTB modulation—transient tight-junction opening using an FSH mutant–occludin peptide conjugate has been shown to reversibly disrupt the BTB without compromising other epithelial barriers (133), and the F5-peptide derived from laminin-γ3 can similarly create a therapeutic window for drug entry (134). Such approaches, however, require rigorous safety evaluation regarding germinal epithelium integrity.

In summary, the BTB should not be treated merely as a passive obstacle to be “crossed,” but rather as a central determinant of dosing strategy design. Future LOH clinical trials must incorporate BTB penetrance as a drug-selection criterion, a principle already established in male contraceptive and infertility drug development (134), and establish interstitial concentration–effect relationships in preclinical models.

### 5.3 Cross-talk with the hypothalamic–pituitary–gonadal axis: a systemic perspective on EV network repair

The decompensation of the LCs–tMacs EV network is not an isolated event, but engages in profound bidirectional cross-talk with the hypothalamic–pituitary–gonadal (HPG) axis. Understanding this interplay is essential for designing systemic intervention strategies centered on the concept of “endocrine network resilience.”

A core vicious cycle operates in the bidirectional regulation between the EV network and the HPG axis: declining LC function leads to reduced testosterone secretion, which, through diminished negative feedback, triggers a compensatory elevation of pituitary LH. However, persistently high LH levels exert an “excitotoxicity” effect on LCs. Beattie et al. directly demonstrated that LH stimulation induces a significant increase in ROS production in primary rat LCs, with aged cells exhibiting a higher ROS peak, prolonged recovery time, and more extensive DNA damage (135). LH overactivates steroidogenesis through the cAMP–PKA pathway, exacerbating the load on the mitochondrial respiratory chain and ROS generation, thereby further aggravating the mitochondrial quality crisis. Simultaneously, the sensitivity of aged LCs to LH is diminished due to receptor downregulation (25), creating a paradoxical state of “elevated LH yet reduced responsiveness.” At the molecular level, progressive accumulation of cGMP signaling in aged LCs is significantly correlated with mitochondrial dysfunction and the downregulation of PGC-1α (Ppargc1a), with cGMP serving as one of the downstream second messengers of LH/hCG stimulation. On the donor side, PGC-1α in aged MHCII^+^ tMacs has lost its responsiveness to AMPK and SIRT1 signaling (44)—meaning that even if enhanced LH signaling indirectly amplifies the “demand signals” from LCs, donor macrophages cannot be effectively activated to increase the supply of healthy mitochondria.

The network repair effects of existing endocrine therapies merit reappraisal. hCG therapy—a classical clinical regimen for stimulating endogenous testosterone secretion—not only promotes steroidogenesis by activating LH receptors on LCs but may also partially restore donor-side function by upregulating PGC-1α (this remains a prospective hypothesis, currently lacking direct experimental evidence). SERMs (e.g., clomiphene) elevate endogenous LH and FSH levels by antagonizing hypothalamic–pituitary estrogen negative feedback, thereby indirectly stimulating LC function. It has been shown that multiple SERMs can directly promote an M2 anti-inflammatory macrophage phenotype by inhibiting the NF-κB pathway, and that their anti-inflammatory effects converge on the downregulation of IL-1β. Whether SERMs can indirectly improve the EV network by modulating tMacs polarization remains an intriguing direction for future exploration. However, the paradox of exogenous testosterone replacement therapy (TRT) cannot be overlooked: although TRT can rapidly correct systemic testosterone deficiency symptoms, it suppresses endogenous LH through negative feedback, which may further weaken the functional reserve of LCs and the compensatory capacity of the EV network (136). A sequential treatment strategy may therefore represent a superior alternative—short-term administration of hCG or SERMs to stimulate endogenous LC function and network repair, followed by a transition to low-dose TRT for maintenance once network efficiency has been partially restored, combined with NAD^+^ precursor supplementation to consolidate network repair (137). Within the network repair framework proposed herein, a “rebuild–consolidate–maintain” three-phase strategy may be envisaged sequentially, commencing with hCG or SERM-mediated activation of the endogenous network, proceeding to low-dose TRT for symptomatic maintenance, and culminating in NAD^+^ precursor supplementation for metabolic consolidation. This conceptual regimen holds theoretical potential for balancing symptomatic relief with etiological repair.

## 6 Conclusions and future perspectives

This review systematically elaborates a theoretical framework positing that the extracellular vesicle–mediated bidirectional mitochondrial transfer network between LCs and testicular macrophages serves as a critical hub for maintaining LC homeostasis. We propose a “three-dimensional decompensation” model whereby aging systematically impairs network efficiency through supply-side collapse, clearance-side obstruction, and communication decoupling. By introducing the concept of a “mitochondrial renewal rate threshold,” this framework further explains how network decompensation triggers a mitochondrial quality crisis, steroidogenic enzyme dysfunction, and a cascade of cell death, culminating in the irreversible collapse of testosterone synthesis. This mechanistic paradigm not only elucidates clinical conundrums of late-onset hypogonadism—including individual variability in susceptibility, progressive symptom worsening, and the limited efficacy of hormone replacement therapy—but also advances the therapeutic paradigm from symptomatic management toward network repair. Nevertheless, several critical limitations must be acknowledged. First, the majority of mechanistic data derive from rodent models; significant interspecies differences in testicular macrophage subpopulations, extracellular vesicle molecular signatures, and network architecture necessitate cautious extrapolation to humans. Second, direct evidence for extracellular vesicle–mediated mitochondrial transfer in the aging human testis remains absent, requiring validation in human tissue specimens. Third, all proposed network-repair strategies—including TREM2 agonists, young decellularized testicular extracellular matrix hydrogels, mesenchymal stem cell–derived extracellular vesicles, and NAD^+^ precursors—remain at the preclinical stage, facing formidable translational challenges such as blood–testis barrier penetration, long-term safety, and individualized dosing regimens. Fourth, the “decompensation threshold” remains a conceptual construct; the diagnostic sensitivity, specificity, and predictive value of candidate biomarkers such as INSL3, extracellular vesicle–miRNA profiles, and mitochondrial DNA mutation burden require prospective cohort validation, and the relative quantitative contributions of extracellular vesicle network decline versus hypothalamic–pituitary–gonadal axis aging to late-onset hypogonadism pathogenesis remain undefined. Looking ahead, a precision diagnostic and therapeutic system grounded in extracellular vesicle network theory holds promise as a novel paradigm for managing this disorder: liquid biopsies may capture early signals of declining network efficiency to identify high-risk individuals during the compensated phase, while multi-target combinatorial interventions may repair three-dimensional network damage. As live imaging, organoid models, and single-cell technologies converge, research on male reproductive aging is transitioning from descriptive science toward mechanism-driven translational medicine. Yet human validation of the network architecture, elucidation of its interplay with other intracellular quality control systems, and resolution of delivery bottlenecks in clinical translation remain critical scientific gaps demanding urgent attention.

## Glossary

3β-HSD1: 3β-hydroxysteroid dehydrogenase type 1
AMPK: AMP-activated protein kinase
ATP: adenosine triphosphate
CCL2: C-C motif chemokine ligand 2
CD38: cyclic ADP-ribose hydrolase
COX-2: cyclooxygenase-2
CYP11A1: cytochrome P450 cholesterol side-chain cleavage enzyme
Drp1: dynamin-related protein 1
ER: estrogen receptor
ESCRT: endosomal sorting complexes required for transport
GCN5: general control non-derepressible 5
HIF-1α: hypoxia-inducible factor 1 alpha
ICD: intracellular domain
IL: interleukin
ITAM: immunoreceptor tyrosine-based activation motif
LC: Leydig cell
LOH: late-onset hypogonadism
MHCII: major histocompatibility complex class II
MMP: mitochondrial membrane potential
mtDNA: mitochondrial DNA
NF-κB: nuclear factor kappa-light-chain-enhancer of activated B cells
NOX4: NADPH oxidase 4
OMM: outer mitochondrial membrane
PGC-1α: peroxisome proliferator-activated receptor gamma coactivator 1-alpha
QPCT/L: glutaminyl-peptide cyclotransferase
RhoA: Ras homolog family member A
SASP: senescence-associated secretory phenotype
sEV: small extracellular vesicle
SIRT: sirtuin
StAR: steroidogenic acute regulatory protein
TFEB: transcription factor EB
TREM2: triggering receptor expressed on myeloid cells 2
TSG101: tumor susceptibility gene 101
VPS4: vacuolar protein sorting 4
8-OHdG: 8-hydroxy-2′-deoxyguanosine
AP2: adaptor protein 2
BTB: blood-testis barrier
CD: cluster of differentiation
CD47: cluster of differentiation 47
CTF: C-terminal fragment
CYP17A1: cytochrome P450 17α-hydroxylase/17,20-lyase
dTECM: decellularized testicular extracellular matrix
ERK: extracellular signal-regulated kinase EV, extracellular vesicle
GSH: glutathione
HPG: hypothalamic-pituitary-gonadal
IFN-γ: interferon-gamma
ILV: intraluminal vesicle
ITGβ1: integrin beta-1
LH: luteinizing hormone
MAPK: mitogen-activated protein kinase
MICOS: mitochondrial contact site and cristae organizing system
MnSOD: manganese superoxide dismutase
MVB: multivesicular body
NLRP3: NLR family pyrin domain containing 3
NR: nicotinamide riboside
OPA1: optic atrophy 1
PI3K: phosphoinositide 3-kinase
RAB27A: Ras-related protein Rab-27A
ROCK: Rho-associated coiled-coil containing protein kinase
SC: Sertoli cell
SHBG: sex hormone-binding globulin
SLC: stem Leydig cell
Syk: spleen tyrosine kinase
tMac: testicular macrophage
TRT: testosterone replacement therapy
VCAM1: vascular cell adhesion molecule 1
ADAM17: A disintegrin and metalloproteinase 17
AR: androgen receptor
cAMP: cyclic adenosine monophosphate
CD206: mannose receptor C-type 1
CME: clathrin-mediated endocytosis
CXCL10: C-X-C motif chemokine ligand 10
DAMP: damage-associated molecular pattern
ECM: extracellular matrix
ERRα: estrogen-related receptor alpha
FSH: follicle-stimulating hormone
hCG: human chorionic gonadotrophin
HRS: hepatocyte growth factor-regulated tyrosine kinase substrate
IGF-1: insulin-like growth factor 1
INSL3: insulin-like factor 3
JNK: c-Jun N-terminal kinase
LMP: lysosomal membrane permeabilization
Mfn1/2: mitofusin ½
miRNA: microRNA
MSC: mesenchymal stem cell
NAD⁺: nicotinamide adenine dinucleotide
NMN: nicotinamide mononucleotide
NRF-1: nuclear respiratory factor 1
OXPHOS: oxidative phosphorylation
PKA: protein kinase A
RCS: respiratory chain supercomplex
ROS: reactive oxygen species
SERM: selective estrogen receptor modulator
SHP-1: Src homology region 2 domain-containing phosphatase-1
SOD2: superoxide dismutase 2
TFAM: transcription factor A, mitochondrial
TNF-α: tumor necrosis factor alpha
Trx2: thioredoxin 2
VEGF: vascular endothelial growth factor
